# Daunorubicin and its hydroxy metabolite in cardiomyocytes: insights into cellular kinetics, toxicity, DNA damage, and dexrazoxane-induced cardioprotection

**DOI:** 10.1007/s00204-025-04095-z

**Published:** 2025-06-07

**Authors:** Lenka Applová, Paulína Dudášová-Hatoková, Jan Kubeš, Nela Váňová, Veronika Keresteš, Adam Reguli, Anna Jirkovská, Jaroslav Roh, Martin Štěrba, Petra Štěrbová-Kovaříková, Tomáš Šimůnek

**Affiliations:** 1 Faculty of Pharmacy in Hradec Králové, Department of Biochemical Sciences, Charles University, Akademika Heyrovského 1203, 500 05 Hradec Králové, Czech Republic; 2 Department of Pharmaceutical Chemistry and Pharmaceutical Analysis, Faculty of Pharmacy in Hradec Králové, Charles University, Hradec Králové, Czech Republic; 3 Department of Organic and Bioorganic Chemistry, Faculty of Pharmacy in Hradec Králové, Charles University, Hradec Králové, Czech Republic; 4 Faculty of Medicine in Hradec Králové, Department of Pharmacology, Charles University, Hradec Králové, Czech Republic

**Keywords:** Daunorubicin, Daunorubicinol, Anthracycline cardiotoxicity, Dexrazoxane protection, DNA damage

## Abstract

Anthracycline anticancer agents, such as daunorubicin and doxorubicin, rank among the most effective and widely used anticancer drugs. However, their benefit is markedly reduced by the risk of severe cardiotoxicity. Anthracyclines undergo metabolic reduction of the side chain carbonyl group, producing hydroxy metabolites implicated in the cardiotoxicity. This study investigated toxicity, metabolism and cellular disposition of daunorubicin and its hydroxy metabolite, daunorubicinol, in isolated rat neonatal cardiomyocytes. Daunorubicin induced concentration-dependent cytotoxicity, whereas the toxicity of exogenously administered daunorubicinol was significantly lower despite induction of similar DNA damage. UHPLC-MS analyses revealed that daunorubicin rapidly penetrates cardiomyocytes and is metabolized to daunorubicinol, which is then released from the cells. The intracellular concentration of daunorubicinol was consistently lower than that of daunorubicin, indicating a reduced tendency for daunorubicinol to accumulate in cardiomyocytes. P-glycoprotein 1 has been shown to actively facilitate the efflux of both daunorubicin and daunorubicinol from cardiomyocytes. Dexrazoxane, the only approved agent for anthracycline cardiotoxicity prevention, did not affect the cellular metabolism or disposition of daunorubicin or its hydroxy metabolite, but it effectively reduced not only daunorubicin-induced cardiotoxicity, but also provided protection against the lower toxicity of daunorubicinol. Moreover, dexrazoxane reduced DNA damage induced by both daunorubicin and its hydroxy metabolite. These findings suggest that daunorubicin is the primary driver of cardiomyocyte cytotoxicity, while its hydroxy metabolite, daunorubicinol, plays a more limited role, challenging the notion that it serves as a significant toxic reservoir.

**Supplementary Information:**

The online version contains supplementary material available at 10.1007/s00204-025-04095-z.

## Introduction

**Fig. 1:**
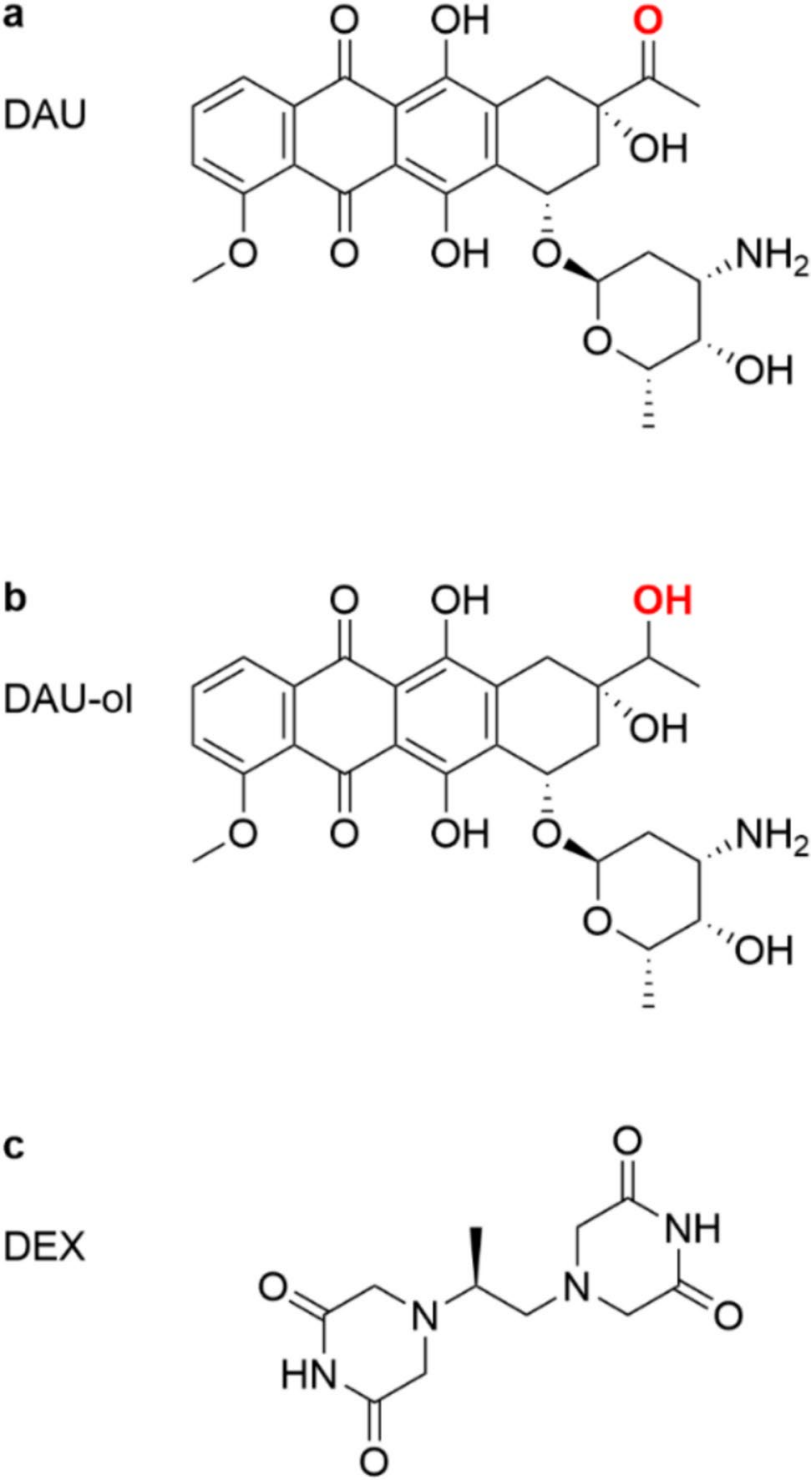
Chemical structures of daunorubicin (**a**; DAU), daunorubicinol (**b**; DAU-ol) and dexrazoxane (**c**; DEX)

Anthracycline (ANT) antineoplastic antibiotics, such as daunorubicin (DAU, Fig. 1a) doxorubicin (DOX), and epirubicin, rank among the most effective and widely used anticancer drugs. Their introduction into clinical practice has led to significant advancement, particularly in pediatric oncology, where they have contributed to notable therapeutic success. Despite being in use for more than half a century, the ANTs continue to be a cornerstone of contemporary combination chemotherapy regimens (Booth et al. 2022). Rather than being replaced by novel targeted agents, there is a tendency to combine ANTs in various schedules to maximize the therapeutic response (Petrelli et al. 2012).

While ANTs are associated with common acute side effects (generally reversible and manageable) typical of classical anticancer therapies, the most significant concern is their potential to cause severe cardiotoxicity (Al-Otaibi et al. 2022; Dempke et al. 2023; Suter and Ewer 2013). The chronic and delayed types of ANT-induced cardiotoxicity are irreversible, resulting in cardiomyopathy and heart failure (Booth et al. 2022; Cvetkovic and Scott 2005; Dempke et al. 2023; Sterba et al. 2013). In addition to overt cardiotoxicity, subclinical myocardial damage may significantly increase cardiovascular morbidity and mortality in long-term cancer survivors (Leger et al. 2015; Menna et al. 2012; Saleh et al. 2021).

The mechanisms underlying ANT-induced cardiotoxicity have been subjected to extensive research yet remain a subject of ongoing debate. For decades, the prevailing hypothesis focused on iron-mediated reactive oxygen species (ROS) generation by ANTs (Keizer et al. 1990; Simunek et al. 2008). Highly reactive hydroxyl radicals generated through this pathway can cause damage to proteins, lipids and DNA leading to myocyte degradation. Cardiac mitochondria are suggested to be closely connected to these damages via high affinity of ANT for cardiolipin in inner membrane leading to ANT cumulation (Booth et al. 2022; Jong et al. 2022; Sawicki et al. 2021). However, recent studies suggest that ROS generation may be a secondary event, with the primary insult involving the interaction between ANTs and topoisomerase IIβ (TOP2B) in cardiomyocytes (Al-Otaibi et al. 2022; Dempke et al. 2023; Vejpongsa and Yeh 2014; Zhang et al. 2012). TOP2B plays a critical role in transcriptional regulation and genome organization (Austin et al. 2018; Vavrova and Simunek 2012), and knockout studies in murine models have demonstrated that deletion of this enzyme in cardiomyocytes can protect the heart from DOX-induced cardiotoxicity (Zhang et al. 2012).

The principal biotransformation pathway of ANTs is two-electron reduction at the C-13 carbonyl group that results in the formation of secondary alcohol metabolites (Forrest et al. 2000; Minotti et al. 2004). This is mediated by a heterogenous family of cytosolic NADPH-dependent carbonyl (CBR) and aldo–keto (AKR) reductases, (collectively referred as carbonyl-reducing enzymes, that catalyze the formation of daunorubicinol (DAU-ol, Fig. 1b), doxorubicinol (DOX-ol), epirubicinol and idarubicinol from their parent drugs. The AKRs have been shown as the primary enzymes involved in DOX reduction in the human heart, whereas the CBRs play a more prominent role in the metabolic conversion of DAU (Mordente et al. 2003).

These hydroxy metabolites have been repeatedly implicated as the major contributors to ANT-induced cardiotoxicity (Al-Otaibi et al. 2022; Menna et al. 2008; Minotti et al. 2004; Olson et al. 1988; Reis-Mendes et al. 2015), and have also been associated with diminished antitumor efficacy (Ax et al. 2000; Minotti et al. 2004; Veitch et al. 2009). The polymorphism in the CBR3 gene with increased formation of ANT metabolites has been associated with a higher propensity of patients for cardiotoxicity development (Bhatia 2020) and likewise, overexpression of human CBRs in the animal hearts resulted in increased cardiotoxicity burden (Forrest et al. 2000). The hydroxy metabolites of ANTs are slightly more polar than their parent drugs, leading to speculations that they may accumulate within cardiomyocytes due to impaired efflux options, and thus causing the chronic cardiotoxicity development (Al-Otaibi et al. 2022; Gianni et al. 2008; Menna et al. 2012, 2008; Mordente et al. 2015; Olson et al. 1988). However, under physiological pH, majority of both ANTs and their hydroxy metabolites will be ionized on common basic daunosamine part of the molecule, whose p*K*_a_ is the same for both ANTs and their respective hydroxy metabolites (Matyjaszczyk et al. 2017). Moreover, the unionized fraction of ANTs and their respective hydroxy metabolites will share very similar physico-chemical properties, with calculated lipophilicity difference between ANT and its respective hydroxy metabolite in tenths to hundredths of logP value (Bavlovic Piskackova et al. 2021b). Therefore, the major differences in the physico-chemically driven processes like permeation through membranes cannot be expected. In fact, conflicting data regarding the exact role of these metabolites in cardiotoxicity have emerged in the literature (Bains et al. 2013; Berg et al. 1999; Bernardini et al. 1991; Forrest et al. 1998; Platel et al. 2001; Zeng et al. 2019). Furthermore, there is significant variability among individual ANTs in terms of their metabolism by these reductases, and this variability is further complicated by interspecies differences in reductase activity.

This study is aimed to contribute to the understanding of the role of hydroxy metabolites in the development of ANT-induced cardiotoxicity. Specifically, we examined and compared the cardiotoxic effects of DAU and DAU-ol and investigated their cellular kinetics and disposition using the primary cultures of isolated rat neonatal ventricular cardiomyocytes (NVCM). We focused on their intracellular accumulation, influx, efflux, and the role of active transport. Toxicity assessments, considering both concentration- and time-dependent effects, were performed in conjunction with precise UHPLC-MS assays of extra- and intra-cellular concentrations of DAU and DAU-ol. In the case of the hydroxy metabolite, we aimed to study both exogenously added DAU-ol (“DAU-ol (EX)”) and DAU-ol endogenously/enzymatically formed by cardiomyocytes (“DAU-ol (ED)”). Additionally, we aimed to explore how these toxicity mechanisms align with the current TOP2B-centered understanding of ANT-induced cardiotoxicity and to assess the effects of the clinically approved cardioprotective agent dexrazoxane (DEX, Fig. 1c).

## Materials and methods

### Chemicals

Dulbecco’s modified Eagle’s medium (DMEM) with nutrient mixture F-12 (DMEM/F-12), horse serum, fetal bovine serum, penicillin/streptomycin solution (5000 U/mL) and sodium pyruvate solution (100 mM) were purchased from Lonza (Belgium). The sera were heat-inactivated prior to use (56 °C for 30 min). Pancreatin, nicotinamide adenine dinucleotide, dimethylsulfoxide, potassium phosphate, Triton X-100, DTT, Tris–HCl buffer, and EDTA were from Sigma-Aldrich/Merck/(USA). Collagenase type II was from Gibco/ThermoFisher Scientific/(USA). Sytox Green nucleic acid stain and SYBR Safe were from Invitrogen-Molecular Probes/ThermoFisher Scientific/(USA), and elacridar (ELA) from MedchemExpress (USA). Daunorubicin hydrochloride and dexrazoxane were obtained from Euroasian Chemicals Pvt. Ltd. (India), daunorubicinol hydrochloride/DAU-ol (EX)/, and internal standards for UHPLC-MS assays (daunorubicin‑^13^C,d_3_ and daunorubicinol‑^13^C,d_3_) were purchased from Toronto Research Chemicals (Canada). Other chemicals used for experiments and analyses (e.g., constituents of buffers) were purchased from Sigma-Aldrich/Merck/(USA) or Penta (Czech Republic).

### Cardiomyocyte isolations

The hearts from 1 to 3-day-old Wistar rats were extracted, minced, and serially digested with the mixture of collagenase and pancreatin. The cells from the supernatants were collected by centrifugation (300 g, RT, 5 min), resuspended in DMEM/F-12 (4 mM sodium pyruvate, 10% fetal bovine serum, 5% horse serum, 1% penicillin/streptomycin) and two hours later, cardiac fibroblasts attached to the plastic bottom, while cardiomyocytes remained floating in the medium (Simunek et al. 2008; Vavrova et al. 2013). The cardiomyocytes were then seeded on 1% gelatine-coated dishes or plates: 24-well plates for lactate dehydrogenase assay (LDH) and γH2AX experiments (0.4 million cells per well), 96-well plates for nucleic acid staining assay (80 thousand per well), 60 mm Petri dishes for in vitro metabolism assessment (4.8 million per dish) and 6-well plates for caspase activity evaluation (1.6 million cells per well). Before the experiments, the medium was changed to serum and pyruvate-free DMEM/F-12. The Czech Ministry of Education, Youth and Sports and the Charles University Faculty of Pharmacy Animal Care Committee approved and supervised all animal procedures and the preparation of NVCM (Project number MSMT-27872/2022-3).

### Cytotoxicity experiments

Sytox Green nucleic acid dye was used to determine cellular viability/toxicity at a concentration of 3 µM. This dye cannot cross the membrane of living cells and causes no toxicity to NVCM. Upon damage to the cell membrane, it binds to nucleic acids and exhibits > 500-fold fluorescence enhancement. Fluorescence was measured at λ = 490 nm excitation and λ = 520 nm emission wavelengths using the Tecan Infinite 200 M micro-plate spectrophotometer (Tecan, Austria). Fluorescence was measured at the indicated time(s), and to determine the total nucleic acid content per well, all the samples were treated with 8% Triton X-100 (2 h, 37 °C) at the end of the experiment. The various experimental protocols are depicted at the top of the figures, including also particular time(s) of various evaluations*.*

Furthermore, extracellular lactate dehydrogenase (LDH) activity was measured in selected experiments as a complementary method for determining cellular viability (*see Supplementary Data*).

### In vitro metabolism and cellular disposition studies of DAU and DAU-ol in NVCM

The conditions of these experiments (drug concentrations and incubation lengths) were designated and checked to avoid distinct reduction of cellular viability which would otherwise interfere with the interpretation of the data. NVCM seated on 60 mm Petri dishes (4.8 million cells per dish) were treated at 37 °C with either DAU or DAU-ol (EX), both at a 1.2 µM concentration, to determine their concentration profiles inside the cardiomyocytes and in cell culture media over the incubation period of up to 24 h. At selected time intervals, the cells were rinsed twice with phosphate-buffered saline on ice (4 °C), harvested by cell scraping, centrifuged (700 g for 10 min at 4 °C), and the pellets were kept at −80 °C until sample preparation followed by UHPLC-MS analyses. At the same time-points, 10 µL of cell culture medium was mixed with ice-cold methanol, spiked with internal standards, and immediately analyzed by UHPLC-MS. Both compounds were also incubated in a cell-free culture medium (on gelatine-coated dishes) to examine their eventual spontaneous and/or chemically catalyzed degradation in this experiment (Suppl. Fig. S1).

To assess the potential involvement of active transport of the compounds to and from NVCM, the cells were incubated with DAU and DAU-ol (EX) at 37 °C, 4 °C and with P-glycoprotein 1 (P-gp) inhibitor elacridar (ELA). ELA is a well-established inhibitor of MDR1a and MDR1b, and it was selected as the most suitable P-gp inhibitor in our settings after initial toxicity screening of various inhibitors (cyclosporin A, zosuquidar, and ELA) as it showed minimal impact on cardiomyocyte viability after 24 h. The cells were preincubated for 30 min for uptake assessment with ELA (2 µM) followed by DAU or DAU-ol (EX) exposure. For efflux experiments, the cells were preincubated with DAU-ol (EX) (for 24 or 6 h), 2× rinsed with phosphate-buffered saline (4 °C) and incubated with fresh cell media with or without ELA (2 µM). The high toxicity of DAU towards the cells under these conditions precluded carrying out efflux experiment. To investigate the potential influence of DEX on DAU and/or DAU-ol (ED, EX) in vitro cellular metabolism and disposition, the cells were co-incubated with 100 µM DEX and DAU or DAU-ol (EX) for 6 h. The concentrations of both compounds determined inside the cardiomyocytes and in cell culture media were compared to those obtained without the DEX exposure. All samples were analyzed in quadruplicate.

### UHPLC-MS analyses

The harvested NVCM pellets were mixed with 100 µL of water, treated on an ultrasonic bath for 6 min and spiked with isotopically labelled internal standards’ solution (daunorubicin-^13^C,d_3_ and daunorubicinol-^13^C,d_3_). Then 900 µL of chloroform:methanol (4:1, *v/v*) mixture was added, and DAU and DAU-ol were extracted using vortex (5 min, 8–12 × 100 rpm) followed by centrifugation (9500 g, 10 min, 4 °C). The resulting organic layer (700 µL) was collected and evaporated to dryness at 40 °C under a gentle stream of nitrogen. The residue was reconstituted in 200 μL of methanol, filtered (0.22 µm PVDF), and 10 μL of the sample was injected into the column. Ten microliters of cell culture medium were diluted with ice-cold methanol containing the internal standards to the final volume of 500 μL, and 10 μL of the sample was injected into the column. The analysis was conducted on a Kinetex C18 column (100 × 2.1 mm, 1.7 µm) with the same guard column type (Phenomenex, USA). The column was kept at 40 °C, and the autosampler thermostat at 8 °C. Mobile phase A (0.0025% formic acid in water, *v/v*) and mobile phase B (acetonitrile:methanol, 2:1, *v/v*) were mixed in the following gradient: 0.0–4.0 (20–90% B), 4.0–5.0 (90% B), 5.1–6.5 (20% B). The flow rate was set at 0.35 mL/min. An Agilent 1290 Infinity II LC with Triple Quad LC/MS—6470 (Agilent Technologies, USA) equipped with Agilent Jet Stream—Electrospray Ionization operating in a positive ion mode was used. The MS settings were identical as previously reported (Bavlovic Piskackova et al. 2021a). Quantification was performed in the selected reaction monitoring mode as specified in Supplementary Table S1. The data were analyzed using Agilent MassHunter Quantitative Analysis software (Agilent Technologies, USA). The method was validated for DAU and DAU-ol assay in NVCM and the cell medium within the concentration ranges of 0.02–2.00 nmol/pellet and 0.009–1.77 µM, respectively. For details on the method validation, see Supplementary Table S2.

### Inhibition of human recombinant topoisomerase IIβ (TOP2B)

The TOP2B activity assay was performed using a recombinant human TOP2B enzyme (Inspiralis, U.K.) incubated with kDNA as previously described (Jirkovska et al. 2021). The reactions containing an appropriate range of tested concentrations, kDNA and human TOP2B were incubated for 30 min at 37 °C. After stopping the reaction with the addition of gel loading buffer, the samples were stained with SYBR Safe for 20 min and visualized using a Gel Doc EZ with Image Lab software (Bio-Rad, USA). The signal of the treated samples was normalized to the positive control (untreated sample; 100%) present on the same gel. The normalized signal of three independent measurements was then expressed as mean ± SD.

### Immunodetection of γH2AX

After treatment, NVCM were lysed in 2% SDS and incubated at 90 °C for 10 min. Protein concentrations were assessed using the bicinchoninic acid method. Ten micrograms of total protein were loaded on 12% Bio-Rad TGX Stain-free gels, separated by SDS-PAGE (150 V, Bio-Rad Mini-PROTEAN Tetra Cell) and transferred on the nitrocellulose membrane using semi-dry approach (Bio-Rad Transblot Turbo). The proteins were detected by mouse anti-γH2AX (1:5000; ab11174, Abcam, UK) and HRP-labelled anti-mouse IgG (1:40,000; A9044 Sigma Aldrich, USA). The acquired chemiluminescent signal was normalized to total protein content. Four independent measurements were expressed as mean percentage relative to control ± SD.

### Caspase activity measurements

The activity of caspase 3/7 was determined using a commercially available kit (Caspase Glo Assays, Promega, USA) based on its ability to cleave specific amino acid sequences present in the substrate for luciferase. Lysates were diluted to equal protein concentration, which was assessed using the bicinchoninic acid method.

### Data analyses

The statistical and graphical software GraphPad Prism version 9 (GraphPad Software, USA) was used in this study. All data are presented as mean ± SD. Data were subjected to one-way ANOVA with Dunnett’s post-hoc analysis and unpaired parametric *t*-test. Differences between groups were considered statistically significant at a significance level of p ≤ 0.05. All measurements were performed in four or more replicates.

## Results

### Cytotoxic effects of DAU and DAU-ol (EX) on cardiomyocytes.

**Fig. 2:**
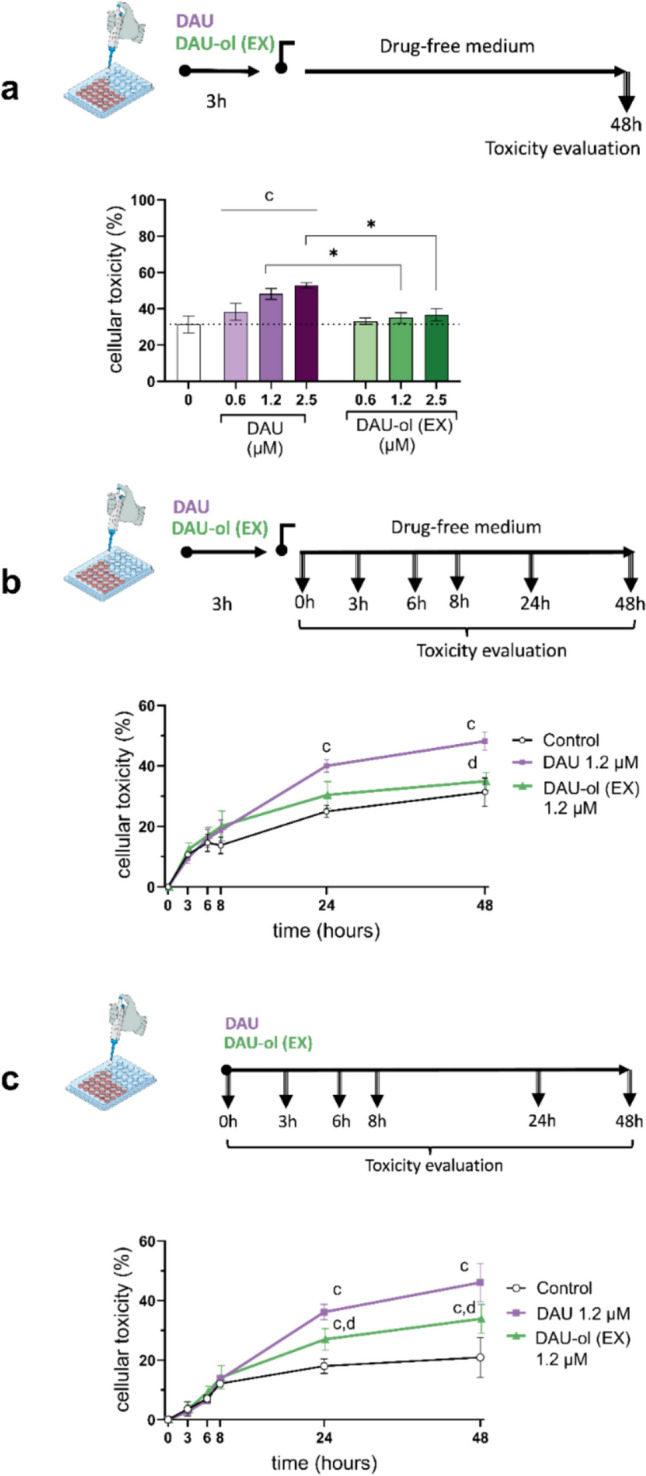
Comparison of cytotoxic effects of daunorubicin (DAU) and exogenously administered daunorubicinol (DAU-ol (EX)) toward rat neonatal ventricular cardiomyocytes (NVCM). **a** NVCM were exposed to 0.6, 1.2, and 2.5 µM DAU or DAU ol (EX) for 3 h, followed by a 48 h drug-free incubation. **b** Time-dependent toxicity assessment of 1.2 µM DAU or DAU-ol (EX) under the same incubation protocol. **c** Continuous 48 h exposure to 1.2 µM DAU and DAU-ol (EX) without wash-out. Nucleic acid stain assay was used for cellular toxicity determinations. Data are presented as means ± SD; n ≥ 4; Statistical significance: one-way ANOVA, p ≤ 0.05 Dunnett test (compared to* c*-control, *d*-daunorubicin) and unpaired parametric t-test (*-between groups)

In the first set of experiments, we compared the cytotoxic effects of various concentrations of DAU and DAU-ol (EX) on NVCM using a previously optimized time protocol and the nucleic staining assay. The NVCM were incubated with DAU or DAU-ol (EX) for 3 h, followed by wash-out and incubation in a drug-free medium until evaluation at 48 h after the treatment. As seen in Fig. 2a, DAU induced concentration-dependent cytotoxicity, with statistical significance achieved in comparison with the control cells at all tested concentrations (0.6, 1.2 and 2.5 µM). At the highest concentration (2.5 µM), DAU induced approximately 22% increase in cardiomyocyte death as compared to the untreated control. In contrast, DAU-ol (EX) exerted no significant toxicity relative to the control group in all concentrations tested. Similar results were obtained in additional experiments when the LDH release assay was used as an alternative method for cytotoxicity determination (Suppl. Fig. S2). The nucleic acid staining assay enabled monitoring of the cytotoxicity over time, and it was therefore performed using both the previously described protocol (Fig. 2b) and the protocol with continuous exposure of the cells to DAU and DAU-ol (EX) without wash-out (Fig. 2c). The cytotoxicity of 1.2 µM DAU increased gradually over time, with significant toxicity achieved, as compared to the control cells, at 24 and 48 h in both incubation protocols. However, 1.2 µM DAU-ol (EX) induced significant toxicity in comparison with the control cells only after continuous exposure (Fig. 2c), yet even in this case, the cytotoxicity found at both 24 and 48 h was significantly lower in comparison with DAU. Overall, these experiments clearly indicated the lower toxicity of exogenously administered DAU-ol to isolated cardiomyocytes compared to DAU.

### Cellular metabolism and disposition of DAU and DAU-ol in cardiomyocytes.

The cardiomyocytes on Petri dishes were first treated with 1.2 µM DAU, and drug concentration profiles of both DAU and DAU-ol (EX) were determined using UHPLC-MS both in cell culture medium and inside the cardiomyocytes. The preliminary experiments aimed at detection of aglycones of both DAU and DAU-ol (ED) during the incubation of the cells with DAU confirmed DAU-ol (ED) as the principal DAU metabolite produced by cardiomyocytes as concentrations of both aglycons were below the lower limits of quantification (*i.e*., 0.009 µM in the cell media or 0.02 nmol/4.8 × 10^6^ cells inside cardiomyocytes). Furthermore, control incubations of both compounds in cell-free media revealed a lack of spontaneous chemical degradation of DAU or DAU-ol (EX) (Suppl. Fig. S1), providing evidence that the observed DAU-ol (ED) formation is a result of metabolic activity of cardiomyocytes.

**Fig. 3:**
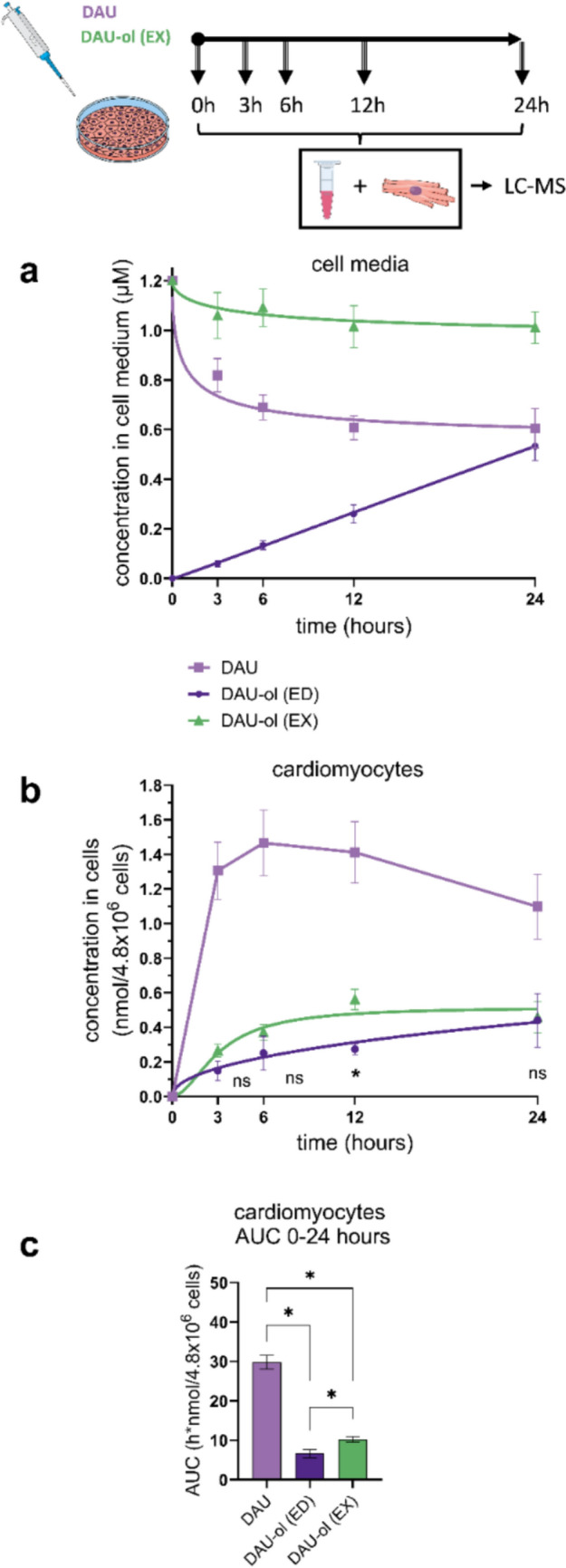
Cellular metabolism and disposition of daunorubicin (DAU) and daunorubicinol (DAU-ol)-either endogenously formed from DAU (DAU-ol (ED)) or exogenously administered (DAU-ol (EX))—in cell culture media and rat neonatal ventricular cardiomyocytes (NVCM)*.* NVCM on petri dishes were incubated with 1.2 µM DAU or DAU-ol (EX) for up to 24 h at 37 °C, and at 0, 3, 6, 12 and 24 h, the drugs’ concentrations in cell media (**a**) and in NVCM (**b**) were analyzed. Areas under the curve were established for all compounds inside the NVCM (**c**). All samples were analyzed by UHPLC-MS. Data are presented as means ± SD; n ≥ 4; Statistical significance: unpaired parametric t-test (*-between groups)

The cell culture media concentration–time profiles determined for DAU and its hydroxy metabolite endogenously formed by the NVCM (DAU-ol (ED)) are presented in Fig. 3a. It shows rather a sharp initial decline in the DAU concentrations in the medium from 1.2 µM to approx. 0.7 µM, indicating relatively fast intracellular penetration of DAU into NVCM. There was only little change in the DAU concentrations from approximately hour 6 onwards suggesting achievement of an equilibrium. This assumption was confirmed by determination of DAU concentrations inside the cardiomyocytes (Fig. 3b), where a steep increase of DAU was detected at the beginning of incubation (3 h), followed by a slight further increase up to 6 h. A subsequent gradual decrease was likely caused by an equilibrium of concentrations between in the intracellular and extracellular compartments and the reduction of DAU to DAU-ol (ED). Concentration–time profile of DAU-ol (ED) in cell culture media during incubation of the cells with DAU (Fig. 3a) showed linear increase up to 24 h, when the concentrations of the metabolite and parent DAU became comparable at approximately 0.6 µM (*i.e.*, half of the initial 1.2 µM DAU concentration). Focusing on the earlier time intervals (up to 120 min) (Suppl. Fig. S3), we detected an increase in DAU-ol (ED) concentrations in the media from 15 th min onward, but during the 1 st hour the concentrations were very still low (≤ 0.01 µM). The concentrations of DAU-ol (ED) determined inside the cardiomyocytes were markedly lower than those of the parent DAU during the whole 24 h experiment (Fig. 3b). This was also apparent from the comparison of the areas under the curves (AUC) of intracellular concentrations from 0 to 24 h (Fig. 3c). These data indicate rapid DAU intake, substantial reductive metabolism and subsequent swift DAU-ol (ED) release from NVCM. Of note, the concentrations of DAU-ol (ED) in media containing living cells were comparable (for up to 120 min) to the concentrations obtained by incubation with free cytosol of NVCM cells (prepared by freeze–thaw method). The latter observation indicated that the cardiomyocyte membrane is not a principial barrier for the appearance of DAU-ol (ED) in cell culture media (Suppl. Fig. S3).

Experiments with cardiomyocytes treated with exogenous DAU-ol (DAU-ol (EX)) showed a much lower rate of decline from the initial 1.2 µM concentration in cell culture media compared to DAU. After 24 h, the DAU-ol (EX) concentration in the cell culture media dropped only to approx. 1.0 µM (Fig. 3a). Nevertheless, the concentrations of DAU-ol (EX) inside the cardiomyocytes were higher for all time points than those determined after the natural metabolic conversion from DAU to DAU-ol (ED) (Fig. 3b). Accordingly, this resulted in significantly higher AUC of intracellular concentrations (0–24 h) of the exogenous DAU-ol compared to the endogenous one (Fig. 3c).

Taken together, these data indicate that (i) cardiomyocytes have a marked ability to reduce DAU to its hydroxy metabolite (DAU-ol (ED)), (ii) DAU-ol can both enter and leave cardiomyocytes, (iii) DAU-ol (ED) achieves lower intracellular concentrations and total exposure than the parent DAU in our experimental conditions, and (iv) exogenously administered DAU-ol (DAU-ol (EX)) penetrates to the cardiomyocytes to achieve higher intracellular concentrations and exposures than are those of metabolically formed DAU-ol (DAU-ol (ED)).

**Fig. 4:**
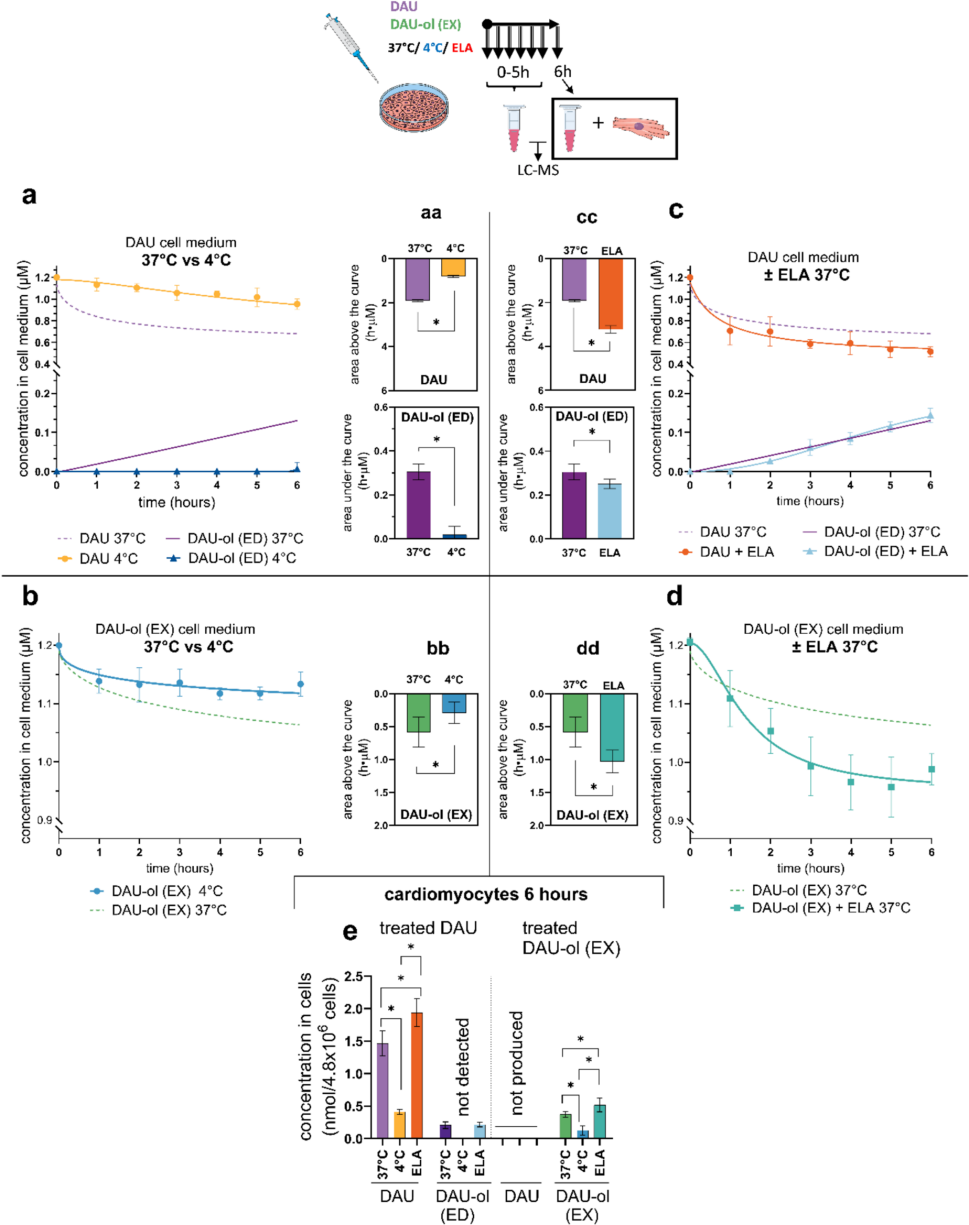
Cellular metabolism and disposition of daunorubicin (DAU) and daunorubicinol (DAU-ol) in rat neonatal ventricular cardiomyocytes (NVCM) at standard (37 °C) and reduced (4 °C) temperature and in the presence of P-gp inhibitor elacridar (ELA). In the case of the metabolite, concentrations of both the endogenously formed DAU-ol from DAU (DAU-ol (ED)) and DAU-ol exogenously administered to cell culture media (DAU-ol (EX)) were studied*.* NVCM on petri dishes were incubated with 1.2 µM DAU or DAU-ol (EX) for up to 6 h at 37 °C (the curves without points for DAU, DAU-ol (ED) and DAU-ol (EX) were used from previous cellular metabolism (Fig. 3)) or 4 °C. The concentration time profiles of DAU, DAU-ol (ED) and DAU-ol (EX) in the cell culture media were determined **a** for DAU, **b** for DAU-ol (EX)). The cumulative decrease of DAU and increase of DAU-ol (ED) concentrations in culture media are expressed as areas above or under the curves (**aa**). **bb** represents the cumulative decrease of DAU-ol (EX) concentrations at 37 °C and 4 °C. The concentration time profiles of DAU, DAU-ol (ED) and DAU-ol (EX) assayed in cell media with 30 min pre-incubation with P-gp inhibitor ELA (2 µM) at 37 °C are shown in panels **c** for DAU and **d** for DAU-ol (EX), respectively. The decrease of DAU and increase of DAU-ol (ED) concentrations in the presence of ELA in culture media are expressed as areas above or under the curves (**cc**). **dd** represents the cumulative decrease of DAU-ol (EX) concentrations at 37 °C or in the presence of ELA. Determinations of concentrations of DAU and DAU-ol inside cardiomyocytes were performed at t = 6 h for all experimental groups (**e**). Data are presented as means ± SD; n ≥ 4. Statistical significance: unpaired parametric t-test (*-between groups)

In further experiments, we wanted to examine whether and to what extent active transport contributes to the intake and efflux of both compounds. Therefore, NVCM were first incubated with 1.2 µM DAU or DAU-ol (EX) at 4 °C for up to 6 h. As seen in Fig. 4a, a decline in DAU concentrations in the cell culture media was also observed at 4 °C, but it was only approximately half of the decrease at the standard 37 °C. This significant difference is also apparent from comparison of the areas above the curves of DAU concentrations in the media up to 6 h (Fig. 4aa). At 4 °C, only negligible levels of endogenous DAU-ol (ED) were determined in the cell media and its corresponding AUC was approximately 15 times lower than under the standard temperature (Fig. 4aa). As seen in Fig. 4e, after the 6 h incubation at 4 °C, a significantly reduced amount of DAU was found inside the cardiomyocytes-less than one third compared to the standard incubation temperature. The latter finding indicated presence of active (temperature-dependent) transport of DAU into the cardiomyocytes. Practically no DAU-ol (ED) could be detected at 4 °C either in the cell culture media (Fig. 4a) or inside the cardiomyocytes (Fig. 4e) due to the apparently suppressed metabolism of DAU to DAU-ol (ED). The cellular toxicity was negligible for both DAU and DAU-ol (EX) under these conditions (Supp. Fig. S4). Significantly reduced decline of DAU-ol (EX) concentrations in cell culture media (*i.e.,* reduced intake to the NVCM) was also observed at 4 °C (Fig. 4b), and this was confirmed by comparison of areas above the curves of DAU-ol (EX) concentrations in the cell media under both temperatures (Fig. 4bb). Correspondingly, significantly lower concentrations of DAU-ol (EX) were determined inside the cardiomyocytes at 4 °C (Fig. 4e).

Based on the results indicating the involvement of the active transport for DAU and/or DAU-ol, we addressed the role of the active efflux transport. In rodents, *MDR1a* and *MDR1b* genes are responsible for the expression of P-gp transporters. ELA was selected as a suitable P-gp inhibitor for these experiments due to its low toxicity in NVCM (for up to 24 h), including the subsequent efflux experiments (Suppl. Figs. S5, S6a,d). Pretreatment of NVCM with ELA (2 µM, 30 min), followed by co-treatment with 1.2 µM DAU, resulted to the higher decline of DAU concentrations in the cell culture media than in the control cells without ELA (Fig. 4c) and correspondingly higher were also areas above the curves (Fig. 4cc). This observation suggested inhibition of efflux transport and reduced DAU recirculation which was further confirmed by the slight but significant increase of DAU content inside the cardiomyocytes at the end of the experiment (Fig. 4e). The ELA treatment had no significant effect on concentrations of endogenous DAU-ol (ED) inside the cells (Fig. 4e), slight but significant difference was observed in AUCs of DAU-ol (ED) concentrations in the cell culture media (0–6 h) (Fig. 4cc). The effect of ELA on concentrations of DAU-ol (EX) in the cell culture media was comparable to that for DAU, *i.e.,* the levels were significantly lower than in cells without ELA (Fig. 4d), with corresponding areas above the curves (Fig. 4dd). This was accompanied by the significant increase of intracellular concentrations of DAU-ol (EX) at the end of the experiment. These results collectively indicate the active involvement of P-gp (and/or other transporter proteins inhibited by ELA, such as the ABCG2/BCRP) in the efflux of both DAU and DAU-ol from the NVCM.

The interpretations of the experiments described above are complicated by the concurrent flow of substances both into and out of cardiomyocytes. To examine efflux only, the NVCM were exposed to 1.2 µM DAU-ol (EX) for 24 h at 37 °C, and after the change of medium to fresh one without DAU-ol (EX), the concentrations of DAU-ol (EX) in the medium and cells were determined by UHPLC-MS. The resulting toxicity of DAU (as compared to the control) was approximately 50% compared to approx. 10% in DAU-ol (EX) (Suppl. Fig. S6b). Therefore, DAU efflux could not be reliably assayed in these conditions as its release from damaged/dead cells would compromise the membrane transport data.

**Fig. 5:**
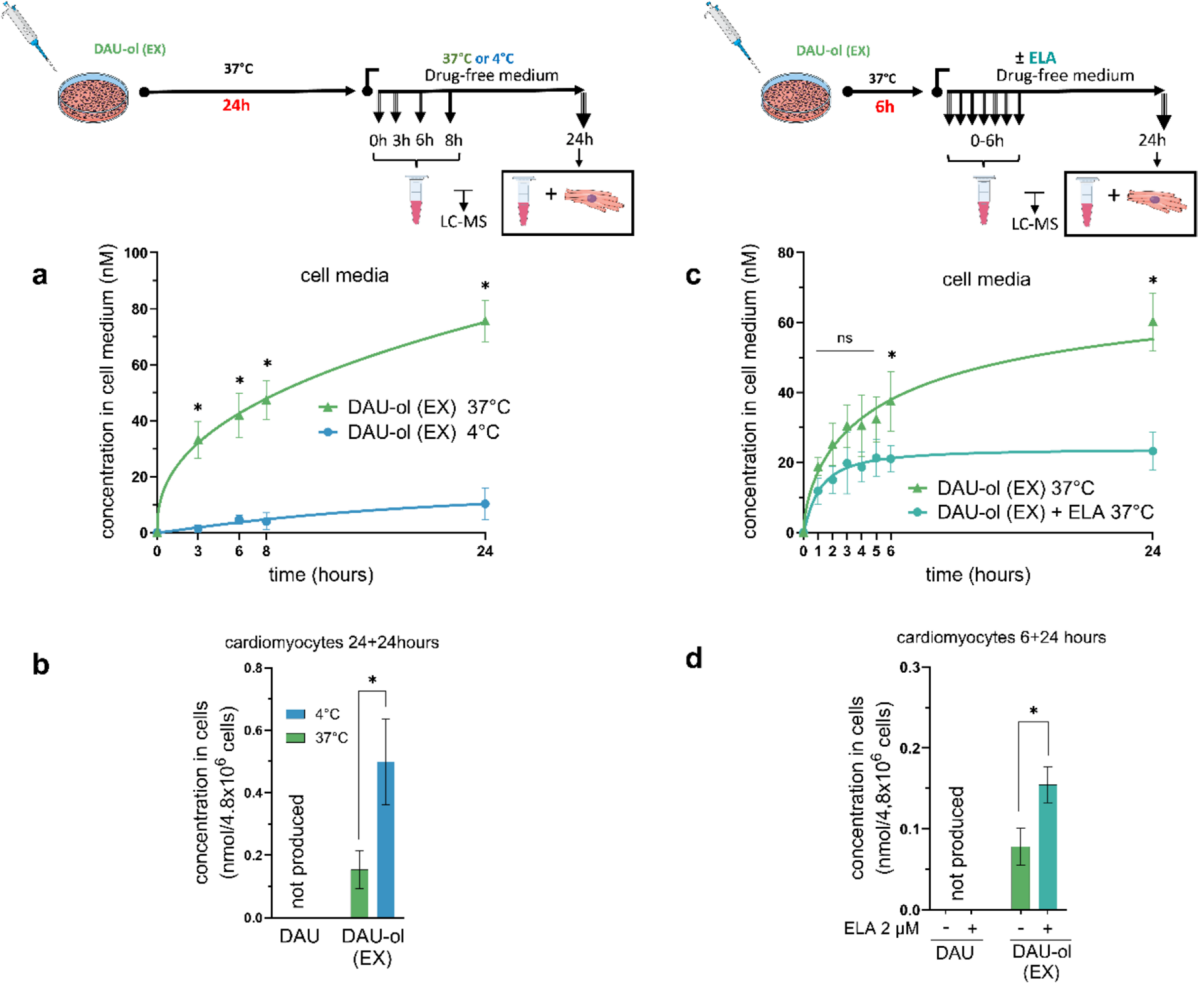
Efflux of exogenously administered daunorubicinol (DAU‑ol (EX)) from rat neonatal ventricular cardiomyocytes (NVCM) to cell culture media. The cardiomyocytes in petri dishes were incubated with 1.2 µM DAU-ol (EX) for 24 h at 37 °C, followed by the 24 h incubations in the drug-free medium at 37 °C or 4 °C (**a, b**). The media samples were assayed by UHPLC-MS at 3, 6, 8, and 24 h after media change **(a)** and concentrations in cells were assayed after 24 h** (b)**. NVCM preincubation with 1.2 µM DAU-ol (EX) for 6 h, followed by incubation for 24 h in the drug-free medium with or without ELA (2 µM), are shown in panel **c** for cell media and panel **d** for NVCM. Data are presented as means ± SD; n ≥ 4. Statistical significance: unpaired parametric t-test (*-between groups)

The release of DAU-ol (EX) from NVCM was first examined at 37 °C and 4 °C. The concentrations of DAU-ol (EX) inside cells were known from previous experiments (Fig. 3b) and confirmed for this set of experiments too. As seen in Fig. 5a, while the concentration of DAU-ol (EX) in the fresh cell culture media increased quickly at 37 °C, its efflux was significantly reduced at 4 °C. This corresponded well with a significantly higher residual amount of DAU-ol (EX) determined inside the cardiomyocytes at the end of the experiment (4 °C vs 37 °C, Fig. 5b). For the subsequent evaluation of DAU-ol (EX) efflux, ELA was used after 6 h preincubation of the cells with DAU-ol (EX) at 37 °C. This incubation time was selected as the compromise between acceptable toxicity (Suppl. Fig. S5) and quantification limits of UHPLC-MS assay in the cells/cell medium. Again, as seen in Fig. 5c, we observed rapid efflux of DAU-ol (EX) from NVCM and its significant (approximately 50%) inhibition by ELA. The analyses of the content of DAU-ol (EX) in the cardiomyocytes at the end of experiment (24 h) confirmed that ELA increased retention of DAU-ol (EX) in the intracellular compartment (Fig. 5d).

### Inhibition of recombinant human topoisomerase IIβ (TOP2B) and DNA damage induction

**Fig. 6:**
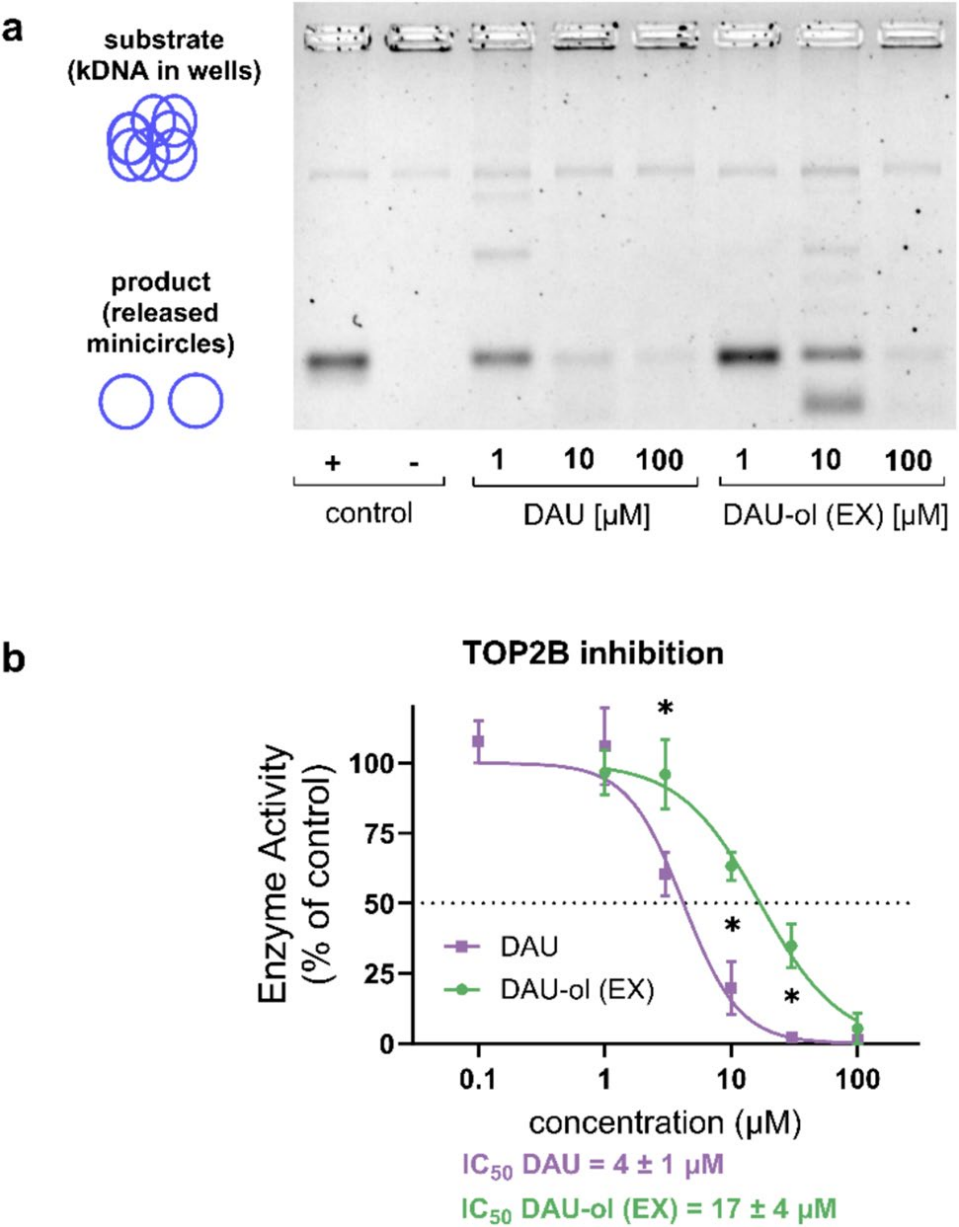
Inhibition of decatenation activity of recombinant human topoisomerase IIβ (TOP2B) by daunorubicin (DAU) and exogenously administrated daunorubicinol (DAU-ol (EX)). The enzyme, catenated DNA (kDNA), and various DAU or DAU-ol (EX) concentrations were incubated for 30 min and the samples were separated on a 1% agarose gel and stained with SYBR Safe. The quantification of visualized gel (**a**) is presented as the inhibition activity of DAU and DAU-ol (EX) toward TOP2B enzyme (**b**). Data are presented as means ± SD; n ≥ 4. Statistical significance: unpaired parametric t-test (*-between groups)

Since TOP2B-mediated DNA damage and subsequent transcriptome changes are currently understood as the key pathogenetic mechanisms of ANT cardiotoxicity, we examined ability of DAU-ol (EX) to inhibit the activity of the recombinant human TOP2B enzyme in the cell-free environment and the results were compared to DAU. As seen in Fig. 6, DAU showed a higher potential to inhibit TOP2B than DAU-ol (EX), with the IC_50_ values being 4 ± 1 µM vs 17 ± 4 µM, respectively.

Furthermore, comet assay was used for DNA damage detection in NVCM incubated for 3 or 6 h with DAU or DAU-ol (EX). This assay, also known as single-cell gel electrophoresis, is a technique used to measure DNA strand breaks in individual cells by observing the migration pattern of DNA fragments under an electric field, which resembles a comet. As seen in Suppl. Fig. S7, both DAU and DAU-ol (EX) displayed a significant, approximately 1.5-fold, increase in comet score over the untreated control that did not differ significantly from each other, indicating similar induction of the DNA damage by DAU and DAU-ol (EX). Additionaly, DNA intercalation activity was compared for DAU and DAU-ol (EX) using an ethidium bromide displacement assay (Suppl. Fig. S8). A slightly lower intercalation activity of DAU-ol (EX) was observed at the lower concentration (10 µM), but no difference between DAU and DAU-ol (EX) was recognizable at 100 µM.

### Effects of DEX on metabolism and cytotoxicity caused by DAU and DAU-ol (EX)

**Fig. 7:**
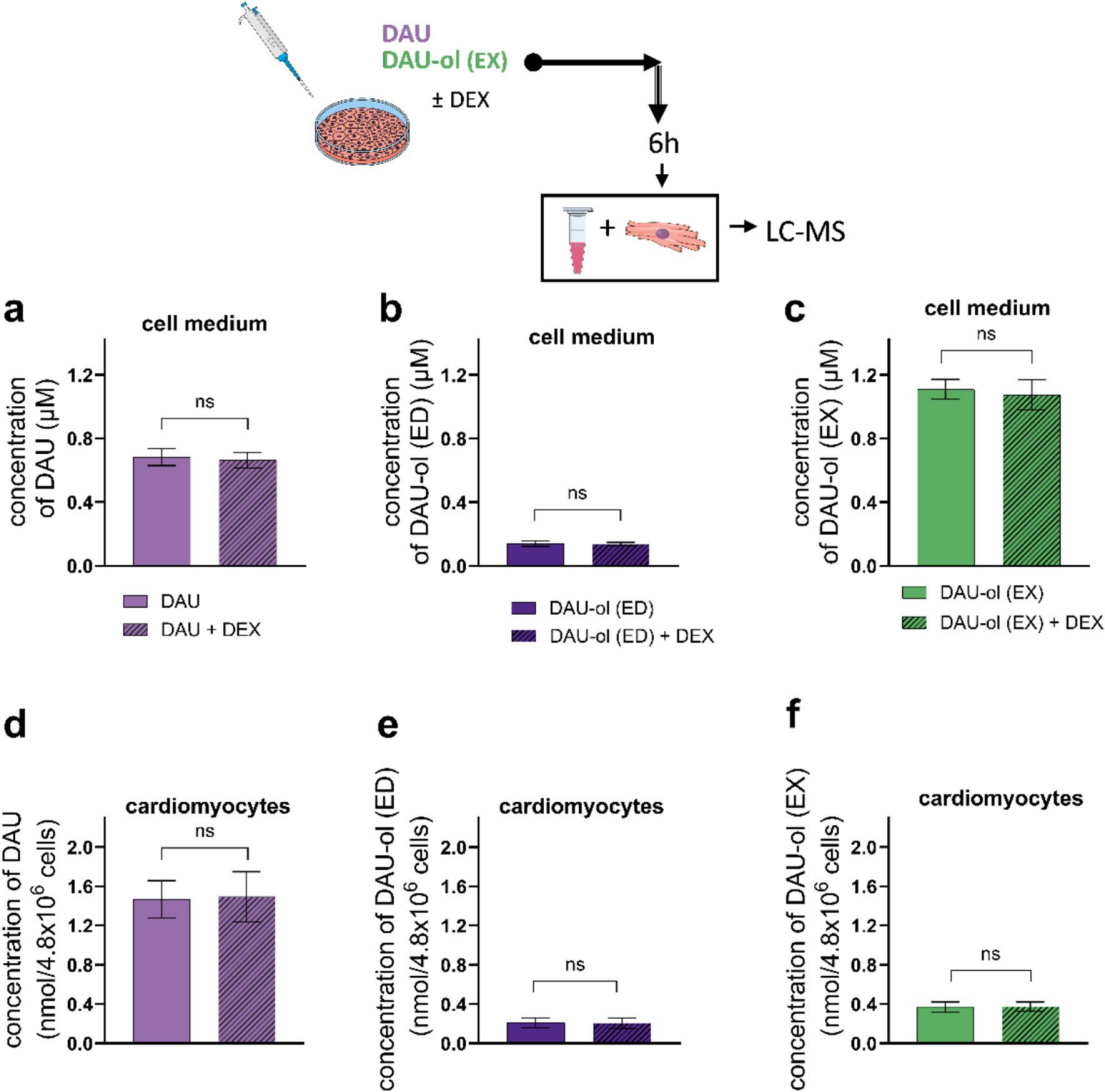
Effect of dexrazoxane co-incubation on cellular metabolism and disposition of daunorubicin (DAU) and daunorubicinol (DAU-ol) in rat neonatal ventricular cardiomyocytes (NVCM) and cell culture media. In the case of the metabolite, both the endogenously formed DAU-ol from DAU (DAU-ol (ED)) and exogenously administered to the cell culture (DAU-ol (EX)) were studied. The cells in petri dishes were co-incubated with 100 µM DEX, 1.2 µM DAU or 1.2 µM DAU-ol (EX) for 6 h. The concentrations of DAU and DAU-ol (ED) after DAU treatment (**a, b**-in cell culture media; **d, e**-in cardiomyocytes) and after DAU-ol (EX) treatment (**c**-in cell culture media and **f**-in cells) were analyzed by UHPLC-MS. Data are presented as means ± SD; n ≥ 4. Statistical significance: unpaired parametric t-test between groups

So far, DEX has been the only protective agent against cardiotoxicity induced by ANTs that has been approved for use in clinical practice. Hence, first we tested its ability to influence the cardiomyocyte metabolism of DAU to DAU-ol (ED) and their cellular disposition. Co-incubation of 100 µM DEX with 1.2 µM DAU did not change the concentrations of DAU or DAU-ol (ED) after 6 h—both in the cell culture media (Fig. 7a,b) or inside the cardiomyocytes (Fig. 7d,e). In addition, there was no effect on the cellular disposition of DAU-ol (EX) (Fig. 7c,f). These results show that DEX does not affect either DAU reductive metabolism or DAU and/or DAU-ol transport through the cardiomyocyte membrane.

**Fig. 8:**
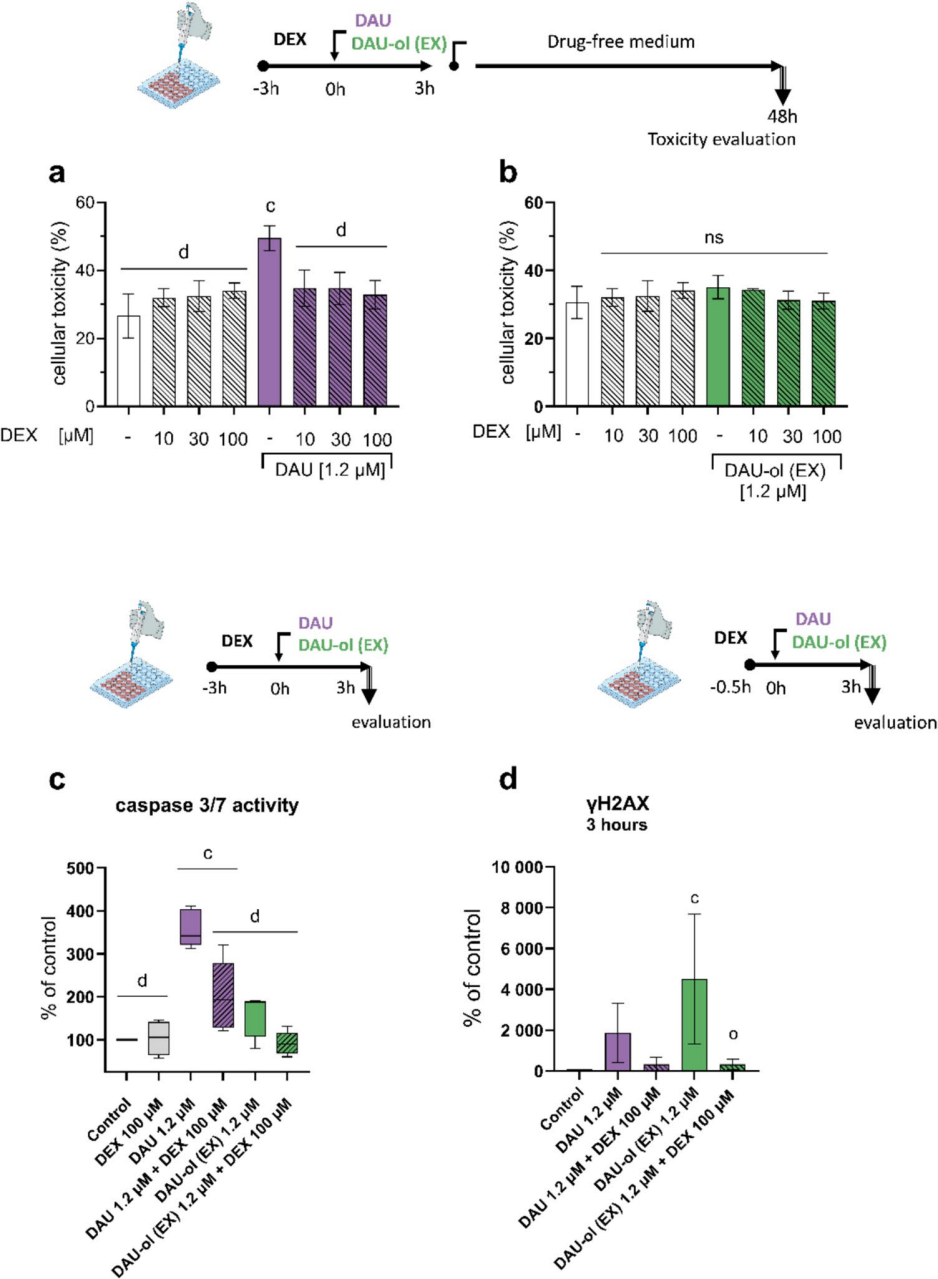
Cytoprotective effect of dexrazoxane (DEX) against toxicity induced by daunorubicin (DAU) or exogenously administrated daunorubicinol (DAU-ol (EX)) in rat neonatal ventricular cardiomyocytes (NVCM). NVCM were preincubated for 3 h with 10, 30 or 100 µM DEX and then incubated with 1.2 µM DAU (**a**) or 1.2 µM DAU-ol (EX) (**b**) for another 3 h with an additional 48-h drug-free period. Toxicity was assessed by nuclear staining assay as an enhanced fluorescent signal in each plate well. Caspase 3/7 activity in cardiomyocytes after 3 h of exposition to 1.2 µM DAU or 1.2 µM DAU-ol (EX) alone or after 3 h of pre-incubation with 100 µM DEX (**c)**. Phosphorylation of γH2AX in NVCM after 30 min pre-incubation with 10 µM DEX followed by the addition of 1.2 µM DAU or DAU-ol (EX) for 3 h is shown in (**d)**. Statistical significance: one-way ANOVA, *p* ≤ 0.05 Dunnett test (compared to* c*-control, *d*-daunorubicin, o-exogenously administered daunorubicinol)

DEX was then assayed for its protective potential against the cytotoxicity of both DAU and DAU-ol (EX). NVCM were preincubated for 3 h with increasing concentrations of DEX (10, 30, 100 µM) followed by 3 h incubation with either DAU or DAU-ol (EX) (both at 1.2 µM). After the medium change, the cells were post-incubated for 48 h in drug free medium and the cytotoxicity was determined by nucleic acid stain. As seen in Fig. 8a, DAU induced significant cellular toxicity, and all tested DEX concentrations provided significant cytoprotection. Using the same incubation protocol, no significant toxicity for DAU-ol (EX) was detected (Fig. 8b). However, when assayed using the alternative incubation protocol with continuous exposure of DAU-ol (EX) for 24 or 48 h, significant toxicity of the DAU-ol (EX) was detected (slightly at 24 h and more distinctly at 48 h) and then DEX was able to dose-dependently restore the viability to the level of untreated control cells (Suppl. Fig. S9).

ANTs are well known for their induction of apoptosis, a form of programmed cell death. Here, the treatment with DAU increased the activity of caspase 3/7 (the key proteases in the execution phase of apoptosis) approximately 3-4 times compared to the control. A slight increase was also visible for DAU-ol (EX), but without significant difference from the control. Pre-treatment with 100 µM DEX for 3 h significantly decreased induction of the caspase activity in DAU, and a trend was also visible in DAU-ol (EX), although it did not achieve statistical significance (Fig. 8c).

The DNA damage, a consequence of the TOP2B poisoning by ANT was also determined by the γH2AX assay, a sensitive method used to detect and quantify DNA double-strand breaks in cells by measuring the specific phosphorylation of the H2AX protein. After 3 h of exposure, an increase in the H2AX phosphorylation was detected for DAU as well as for DAU-ol (EX) (both at 1.2 µM). Pretreatment with 10 µM DEX nearly fully protected the NVCM against the H2AX phosphorylation for both DAU and DAU-ol (EX) (Fig. 8d).

Hence, DEX has been shown to effectively protect the cardiomyocytes not only from the toxicity of DAU, but also from the lower toxicity induced by DAU-ol (EX).

## Discussion

In the present study, using primary neonatal cardiomyocytes, we showed that (i) DAU as well as DAU-ol can enter and leave cardiomyocytes with a substantial contribution of the active transport, (ii) DAU is predominantly reduced to DAU-ol in cardiomyocytes under these conditions, suggesting this may be a major metabolic route, although contributions of other pathways cannot be excluded, (iii) exposure of the intracellular compartment to the endogenously produced DAU-ol is considerably lower than to parent DAU, (iv) exposure of cardiomyocytes to exogenous DAU-ol induces significantly lower cytotoxicity than exposure to parent DAU despite the achievement of the higher intracellular concentrations, (v) the cardioprotectant DEX has no impact on the cardiomyocyte reduction of DAU to DAU-ol or their cellular disposition, (vi) DEX is able to protect cardiomyocytes from the pronounced cytotoxicity of DAU, but also from the lower cytotoxicity of DAU-ol, and (vii) both DAU and DAU-ol can induce DNA damage in cardiomyocytes, which is inhibited by DEX.

To date, the actual involvement, specific role(s), and significance of the hydroxy metabolites of ANTs for ANT cardiotoxicity development has been an unresolved issue. However, in most review papers they have been listed among the plausible mechanisms, or even determinants, of ANT-induced cardiotoxicity (Al-Otaibi et al. 2022; Menna et al. 2012; Minotti et al. 2004). The role of the hydroxy metabolites of ANTs has attracted considerable attention since the 1980 s when series of studies have been published addressing their anticancer action (Olson et al. 1988; Ozols et al. 1980), pharmacokinetics, cardiotoxicity (Del Tacca et al. 1985; Peters et al. 1981), and effects on ion pumps (Boucek et al. 1987; Olson et al. 1988). DOX-ol was reported to have: (i) antitumor activity, although weaker than parent DOX (Ozols et al. 1980; Peters et al. 1981), (ii) cumulation in the heart, albeit slower in comparison to parent DOX (Peters et al. 1981), (iii) higher potential than DOX to inhibit the contraction of the isolated rabbit papillary muscle as well as various ion pumps, such as the calcium pump of sarcoplasmic reticulum, although these were acute functional experiments with concentrations (tens to hundreds of μM) beyond clinical relevance (Boucek et al. 1987; Olson et al. 1988). Eventually, DOX-ol has been called a “highly toxic metabolite” in the influential PNAS paper (Olson et al. 1988), although its own cytotoxicity to cardiomyocytes was not directly demonstrated. Whereas the initial studies mostly focused on the effects of DOX-ol on calcium handling (that may contribute to systolic and/or diastolic dysfunctions), later studies emphasized the iron metabolism disbalance, as DOX-ol can delocalize Fe^2+^ from the [4 Fe-4S] cluster of aconitase and disrupt switch between aconitase and iron regulatory protein IRP-1 (Minotti et al. 1998). However, the role of hydroxy metabolites of ANTs with respect to the TOP2B-mediated DNA damage has not been examined yet.

DAU is an anticancer drug listed on the WHO Model List of Essential Medicines (WHO 2023), usually used for hematological malignancies, particularly acute leukemias. It is generally agreed that DAU shares the mechanisms of action (both anticancer and cardiotoxic) with DOX and other ANTs (McGowan et al. 2017; Minotti et al. 2004). However, reductive metabolization of DAU seems to be generally less variable than that of DOX and DAU also typically undergoes more extensive secondary alcohol production than DOX (Mordente et al. 2003; Qiao et al. 2024; Robert and Gianni 1993). Therefore, DAU can be considered a suitable model ANT drug for studying the role of reductive metabolism in ANT cardiotoxicity, although any generalization (either with respect to the whole ANT class or species) should be treated with caution (Mordente et al. 2003).

Our study is the first to demonstrate that DAU-ol (EX) exhibits significantly lower cytotoxicity toward primary rat neonatal cardiomyocytes compared to DAU, aligning with findings from previous research. In AC16 human differentiated cardiomyocytes, DOX-ol and other metabolites exhibited reduced mitochondrial and lysosomal dysfunction compared to the parent compound, DOX (Reis-Mendes et al. 2024). Studies conducted by (Bains et al. 2013; Zeng et al. 2019) demonstrated that DAU-ol and DOX-ol exhibited lower toxicity compared to the parent compounds DOX and DAU in cardiomyoblast-derived H9c2 cells. However, the latter cells were proliferating and possess considerably more complex molecular phenotype distinct from post-mitotic cardiomyocytes (Lenco et al. 2015), which made the translatability of the previous findings uncertain. Transport experiments performed so far used other cell types than cardiomyocytes and suggested that ANTs enter the cells usually by passive (or facilitated) diffusion and absorptive endocytosis (Huang et al. 2018; Peterson and Trouet 1978; Sasaya et al. 1998). Nevertheless, some studies indicated even a flip-flop phenomenon rather than passive diffusion which was associated with presence of charge on the primary amine of daunosamin under physiological pH (Evans et al. 2009; Regev and Eytan 1997; Regev et al. 2005). At 37 °C, the flip-flop half-life was measured at 0.7 min for DOX and 0.15 min for DAU (Regev and Eytan 1997), which is in good agreement with the relatively fast rate of DAU entry into cardiomyocytes observed in the present study.

After entering the cardiomyocytes, DAU has been in our study shown to quickly and effectively undergo metabolic reduction. However, our data also indicate that endogenously formed DAU-ol plays rather minor role in the cytotoxicity of the parent DAU. This conclusion can be drawn from our observation that incubation of the cells with DAU-ol (EX) induced higher intracellular concentrations (as well as the total intracellular exposures) than DAU-ol (ED) and yet the cytotoxicity of the DAU-ol (EX) was significantly lower than that of DAU. Indeed, if the DAU-ol (ED) would be dominantly responsible for the cytotoxicity induced by treatment of cardiomyocytes with DAU, as it has been repeatedly proposed in the literature (Menna et al. 2008; Minotti et al. 2004; Olson et al. 1988), then the DAU-ol (EX) treatment would have to cause similar or higher cytotoxicity than observed with the parent DAU, which was evidently not the case. However, for detailed interpretation of the toxicity of DAU vs DAU-ol (EX) it is important to note that in our experiments with longer incubation times (≈ hours), the observed effects of administered DAU to cardiomyocytes cannot be attributed solely to DAU itself, but rather to a mixture of DAU and DAU-ol (ED)-in variable and continuously changing ratio.

P-gp is a membrane-bound efflux transporter, which plays a crucial role in modulating cellular exposure to xenobiotics and is therefore a key factor in the assessment of drug-induced toxicity. In a study investigating the expression and localization of P-gp in human heart, it was detected in all heart tissue samples tested, predominantly in endothelial cells of capillaries and arterioles and with a wide interindividual variability. Interestingly, reduced expression of P-gp has been shown in patients with dilated cardiomyopathy (Meissner et al. 2002). Other studies, however, suggest that P-gp expression can be detected in cardiomyocytes of diseased models, such as chronic ischemia (Lazarowski et al. 2005), although the pattern is inconsistent. It has been generally accepted that DAU, as well as the other parent ANTs, are substrates of P-gp in both cancer and cardiac cells (Callies et al. 2004; Evans et al. 2009; Nguyen et al. 2020; Regev and Eytan 1997; Uddin et al. 2022). Nevertheless, the hydroxy metabolites of ANTs have been suggested to accumulate in cardiomyocytes, allegedly forming a long-lasting intracellular drug reservoir that could be responsible for chronic or even delayed cardiotoxicity (Del Tacca et al. 1985; Gianni et al. 2008; Menna et al. 2008; Mordente et al. 2003; Olson et al. 1988). On the other hand, DAU-ol has been also mentioned as the possible P-gp substrate, although so far only for cancer cells only (Callies et al. 2003; Schroder et al. 2000) and data for DAU-ol transport in cardiomyocytes were lacking. However, a MDR1a knockout mice showed increased myocardial accumulation of both DOX and DOX-ol, although their plasma pharmacokinetics was changed only slightly (van Asperen et al. 1999), which is in good agreement with our cardiomyocyte data for DAU and DAU-ol (where both MDR1a and MDR1b were inhibited by ELA).

Many previous studies have suggested the inhibition of carbonyl reductases as a possible cardioprotective strategy (Mordente et al. 2001; Sorf et al. 2019). However, our present study does not support this concept. On the contrary, it indicates that although the inhibition of DAU carbonyl reduction could indeed preserve its anticancer efficiency towards cancer cells, it may unfortunately also increase the exposure of the cardiomyocytes to the more cytotoxic parent DAU. Of note, in our previous study, carbonyl reductase inhibition by various flavonoids (assessed by DOX to DOX-ol conversion using HPLC) did not correlate with their cardioprotective efficiency against DOX in NVCM (Kaiserova et al. 2007). In this study, pharmacological inhibition or genetic knockout/silencing of CBR/AKR was not performed due to the extreme redundancy and complexity of these enzyme families. Multiple reductases’ isoforms with overlapping functions, species-specific differences, and compensatory mechanisms could obscure clear conclusions, and inhibition or silencing might unpredictably alter metabolic pathways rather than isolating the specific contribution of DAU-ol formation to cardiotoxicity. While such approaches could be explored in future dedicated studies, our present focus was on characterizing DAU and DAU-ol kinetics and cytotoxicity under endogenous conditions to provide physiologically relevant insights.

Regarding the possible mechanisms of toxicity, we examined ability of both DAU and DAU-ol to intercalate in the DNA, inhibit recombinant TOP2B (in the cell-free decatenation assay) and induce DNA damage (γH2AX induction and the comet assay). The intercalation activity of DAU-ol as compared to DAU was similar or slightly lower depending on the concentrations used, while TOP2B inhibition activity was approximately four-fold lower in DAU-ol as compared with DAU. In light of the increasingly recognized role of TOP2B as the main target and mediator of ANT cardiotoxicity (Vejpongsa and Yeh 2014; Zhang et al. 2012), the lower affinity of DAU-ol to TOP2B may be related to its lower observed cytotoxicity in cardiomyocytes. On the other hand, we did not observe the lower activity of DAU-ol in the DNA damage assays. Therefore, we cannot exclude that, albeit DAU-ol is not considerably cytotoxic on its own, it can contribute to the toxic effects of the parent DAU by augmenting the DNA damage. In this context it is noteworthy that the TOP2B-dependent DNA damage has been proposed as a mandatory but not the exclusive and sufficient mechanism for the cardiotoxicity development and concomitant participation of additional ANT-induced effects (such as the chromatin damage) may be required (Qiao et al. 2020).

We also studied in cardiomyocytes for the first time the interaction of DAU and DAU-ol (EX) with DEX, the only cardioprotective agent approved for clinical use (Jirkovsky et al. 2021). Our results strongly suggest that DEX-induced cardioprotection does not depend on alteration of the metabolism and/or disposition of DAU in the cardiomyocytes. In previous studies, DEX also did not modify the DAU reductive metabolism and/or cellular kinetics of DAU (DOX) or DAU-ol (ED) in different cell lines (Bains et al. 2013; Mordente et al. 2015). In addition, we have for the first time shown that DEX protects not only against the DAU toxicity but also against the (much weaker, but still present) toxicity of DAU-ol (EX) and this was associated with the protection against the γH2AX phosphorylation induced by both DAU and DAU-ol (EX). Hence, if DAU-ol (ED) contributes to the DAU cardiotoxicity through the induction of DNA double-strand breaks, DEX can interfere with this process through the catalytic inhibition of TOP2B.

In conclusion, our study sheds new light on the intracellular kinetics and toxicodynamics of both DAU and its hydroxy metabolite in cardiomyocytes, offering a better understanding of their roles in cardiotoxicity. Our data challenge the prevailing view that DAU-ol serves as a toxic reservoir in cardiomyocytes. Instead, we demonstrate that DAU-ol is rapidly formed, efficiently exported, and significantly less cytotoxic than its parent compound. This suggests that reductive metabolism may act as a detoxification mechanism rather than a driver of cardiotoxicity. These findings might have important implications: therapeutic strategies aiming to inhibit carbonyl reductases could unintentionally increase cardiac exposure to the more toxic parent drug. These findings prompt a reevaluation of metabolic modulation strategies in anthracycline cardioprotection, particularly those aiming to suppress reductive metabolism. Despite the reduced cytotoxicity of DAU-ol compared to DAU, which can be caused by the lower affinity to TOP2B, both compounds induced comparable DNA damage. Furthermore, cardioprotective effects of DEX are not related to alterations in DAU metabolism and disposition in cardiomyocytes, but DEX apparently reduced the significant DNA damage induced by both DAU and DAU-ol in these primary cells. The latter effects may be involved to some extent in the overall cardioprotective benefits provided by DEX. Our findings emphasize the importance of the complex and dynamic interplay between ANT metabolism, intracellular kinetics, and cytotoxicity in cardiomyocytes.

Finally, it is important to acknowledge that NVCM, while widely used in experimental cardiovascular research, have inherent limitations when modeling adult cardiac physiology. NVCM may differ from adult cardiomyocytes in both metabolic profile and in the expression of various proteins (Guo and Pu 2020). Therefore, future studies using adult cardiomyocytes and in vivo models (using both rodents and non-rodent species) will be essential to further validate these observations.

## Supplementary Information

Below is the link to the electronic supplementary material.

Supplementary file1 (PDF 828 KB)

## Data Availability

The raw source data supporting the findings of this study are publicly available on Zenodo at 10.5281/zenodo.15551093

## Abbreviations

AKR: Aldo-keto reductase
ANT(s): Anthracycline(s)
AUC: Area under the curve
CBR: Carbonyl reductase
DAU: Daunorubicin
DAU-ol: Daunorubicinol
DAU-ol (EX): Daunorubicinol exogenously administered to the experiment
DAU-ol (ED): Daunorubicinol endogenously produced by cardiomyocytes from administered daunorubicin
DEX: Dexrazoxane, ICRF-187
DOX: Doxorubicin
DOX-ol: Doxorubicinol
ELA: Elacridar, GF120918
LDH: Lactate dehydrogenase
P-gp: P-glycoprotein 1, also known as multidrug resistance protein 1 (MDR1), or ATP-binding cassette sub-family B member 1 (ABCB1)
NVCM: Neonatal ventricular cardiomyocytes
ROS: Reactive oxygen species
TOP2B: Topoisomerase IIβ
UHPLC-MS: Ultra-high-performance liquid chromatography-mass spectrometry
