# Supplementary material for: Daunorubicin and its hydroxy metabolite in cardiomyocytes: insights into cellular kinetics, toxicity, DNA damage, and dexrazoxane-induced cardioprotection
Source: Arch Toxicol. 2025 Jun 7;99(9):3751–68. doi: 10.1007/s00204-025-04095-z (PMC12408707; doi:10.1007/s00204-025-04095-z)
Supplement: Supplementary file 1 — Supplementary file1 (PDF 828 KB) [file 204_2025_4095_MOESM1_ESM.pdf]

SUPPLEMENTARY MATERIAL

Daunorubicin and its hydroxy metabolite in cardiomyocytes: Insights into cellular kinetics, toxicity, DNA damage, and dexrazoxane-induced cardioprotection

Lenka Applová[1], Paulína Dudášová-Hatoková[2], Jan Kubeš[1], Nela Váňová[2], Veronika Keresteš[1], Adam Reguli[2], Anna Jirkovská[1], Jaroslav Roh[3], Martin Štěrba[4], Petra Štěrbová-Kovaříková[2], Tomáš Šimůnek[1,*]

[1] Department of Biochemical Sciences, Faculty of Pharmacy in Hradec Králové, Charles University, Czech Republic

[2] Department of Pharmaceutical Chemistry and Pharmaceutical Analysis, Faculty of Pharmacy in Hradec Králové, Charles University, Czech Republic

[3] Department of Organic and Bioorganic chemistry, Faculty of Pharmacy in Hradec Králové, Charles University, Czech Republic.

[4] Department of Pharmacology, Faculty of Medicine in in Hradec Králové, Charles University, Czech Republic.

Supplementary Tables

Table S1

Details on selected reaction monitoring used for the assay of daunorubicin (DAU) and daunorubicinol (DAU-ol) in cell culture media and neonatal ventricular cardiomyocytes (NVCM).

Analyte	Precursor ion (*m/z*)	Product ion (*m/z*)	Collision energy (V)
DAU	528.2	363.1*	16
		321.1	36
DAU-ol	530.2	383.1*	12
		321.1	24
DAU-^{13}C,d_3 (*I.S.*)	532.5	367.1*	16
		325.1	32
DAU-ol-^{13}C,d_3 (*I.S.*)	534.6	387.1*	12
		325.1	28

* confirmation product ion

Table S2

Validation of a UHPLC-MS/MS assay for the determination of daunorubicin (DAU) and daunorubicinol (DAU-ol) in cell culture medium and neonatal ventricular cardiomyocytes (NVCM).

Medium

Linearity

Analyte	Range (µM)	Mean equation y =	Mean R^2
DAU	0.009-1.773	1.060648x+0.008660	0.9991
DAU-ol	0.009-1.773	0.841580x+0.008246	0.9989

Added (µM)	Intra-day			Inter-day		
	Accuracy (%)	Precision RSD (%)	ME norm. (%)	Accuracy (%)	Precision RSD (%)	ME norm. (%)
DAU						
0.009	95.8	3.0	-	97.4	1.2	-
0.018	98.6	1.8	106.1	99.5	0.8	107.9
0.177	102.5	1.9	-	101.7	2.2	-
1.773	98.7	0.7	-	98.7	1.0	-
DAU-ol						
0.009	97.6	2.0	-	99.2	1.2	-
0.018	97.1	2.6	101.4	98.3	0.8	109.2
0.177	102.2	1.7	-	102.1	3.2	-
1.773	98.8	0.6	-	98.5	1.4	-

Cardiomyocytes (NVCM)

Linearity

Analyte	Range (nmol/4.8×10^6 NVCM)	Mean equation y =	Mean R^2
DAU	0.02-2.13	0.188x+0.549	0.9993
DAU-ol	0.02-2.13	0.223x+0.610	0.9993

Added (nmol/ 4.8×10^6 NVCM)	Intra-day				Inter-day			
	Accuracy (%)	Precision RSD (%)	Recovery (%)	ME norm. (%)	Accuracy (%)	Precision RSD (%)	Recovery (%)	ME norm. (%)
DAU								
0.02	96.7	2.0	63.0	106.5	97.0	2.4	57.5	102.7
0.71	97.7	2.3	-	-	98.1	2.0	-	-
2.13	94.3	2.6	-	-	94.4	3.7	-	-
DAU-ol								
0.02	96.2	2.6	58.5	102.9	96.8	2.5	54.1	101.8
0.71	96.8	3.1	-	-	98.4	2.8	-	-
2.13	93.0	3.4	-	-	93.1	2.0	-	-

The calibration curves were constructed using a weighted linear regression method applying a weighting factor of 1/x.

Supplementary Figures

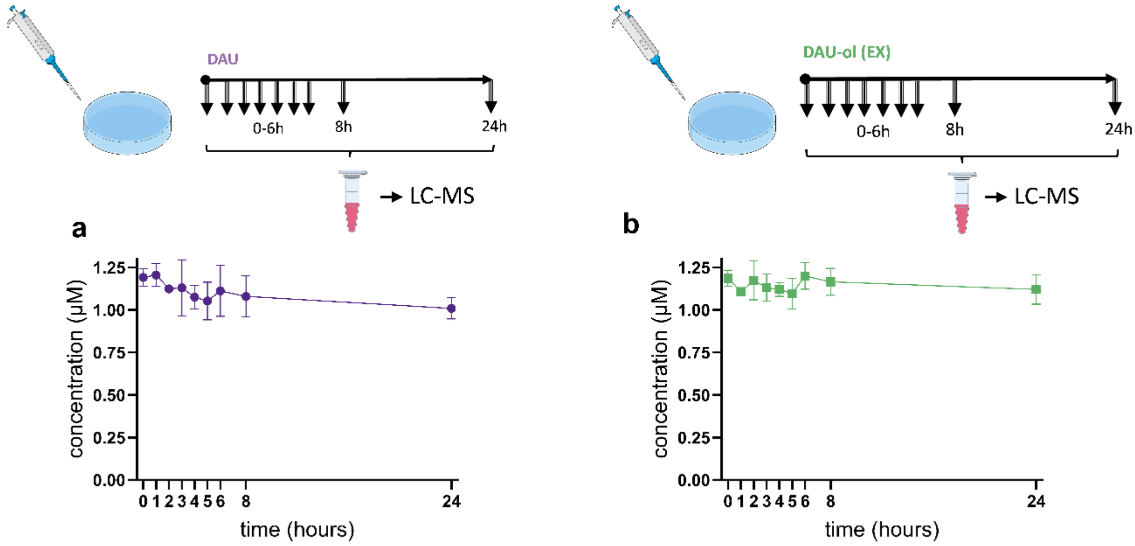

Figure S1. Stability assessments of daunorubicin (DAU) and exogenously administered daunorubicinol (DAU-ol (EX)) in cell free media. The concentrations of DAU (**a**) or DAU-ol (EX) (**b**) were determined in culture media in petri dishes covered with 1 % gelatine, during incubation with 1.2 µM DAU or DAU-ol (EX) without cells. Data are presented as means ± SD ($n \geq 4$). Statistical significance (one-way ANOVA, $p \leq 0.05$; * – between groups).

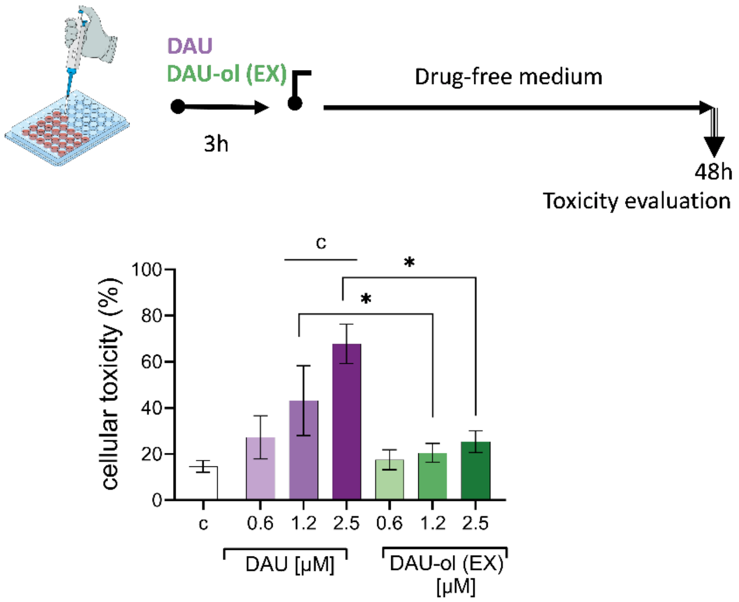

Figure S2. LDH Leakage Assay for additional comparison of daunorubicin (DAU) and exogenously administered daunorubicinol (DAU-ol (EX)) cytotoxic effects toward rat neonatal ventricular cardiomyocytes (NVCM). NVCM were incubated with 0.6, 1.2 and 2.5 µM DAU or DAU-ol (EX) for 3 hours, followed by 48 hours of drug-free incubation. Cellular toxicity was determined using the assay based on measuring extracellular LDH activity as an index of cellular membrane damage (Legrand et al. 1992). The sample of the culture medium was taken from each experimental well, and the activity of LDH was assayed in Tris-HCl buffer (pH 8.9) containing 35 mM lactic acid and 5 mM NAD^+. The rate of NAD^+ reduction was monitored spectrophotometrically at $\lambda = 340$ nm for 2 min at room temperature using a microplate spectrophotometer Tecan Infinite 200 M (Tecan, Austria). To determine the total cellular LDH level, all the wells were treated with lysis buffer (0.1 M potassium phosphate, 1% Triton X-100, 1 mM DTT, 2 mM EDTA, pH 7.8, 30 min at 37 °C). The slope of the linear region was calculated, and the data were expressed as a percentage of the total LDH in each completely lysed well. Data are presented as means ± *SD* ($n \geq 4$). Statistical significance: one-way ANOVA, $p \leq 0.05$ Dunnett test (compared to *c* – control) and unpaired parametric t--test (* – between groups).

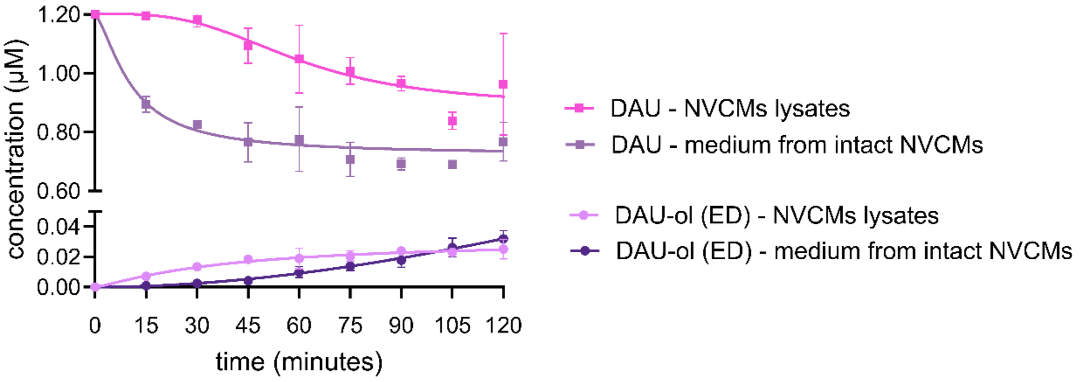

Figure S3. *In vitro* assessment of metabolic activity of intact and lysed rat neonatal ventricular cardiomyocytes (NVCM). The petri dishes containing isolated cardiomyocytes were treated with 1.2 µM DAU, and every 15 minutes, the concentrations of DAU and endogenously formed daunorubicinol (DAU-ol (ED) in cell media were analysed by UHPLC-MS (intact NVCM). In the case of NVCM lysates, the cardiomyocytes on petri dishes were treated with accutase (30 minutes, RT, shaking) to release the cells to the suspension (trypan blue was used to confirm viability). After that, the suspension in NADPH regeneration system (2.54 mM $NADP^+$, 10 mM $MgCl_2$, 18 mM glucose-6-phosphate, and 0.88 U of glucose-6-phosphate dehydrogenase in 0.1 M phosphate buffer, pH 7.4) was frozen at -80 °C for 30 minutes and then thawed at room temperature. This procedure was repeated three times and confirmed by trypan blue visualisation (freeze-thaw method). After the cell membrane rupture, the intracellular matrix in the reaction solution was incubated with 1.2 µM DAU, and again, every 15 minutes, the concentrations of DAU and DAU-ol (ED) were analysed by UHPLC-MS. Data are presented as means ± SD (n = 4 for 60 and 120 minutes, n = 2 for other time points).

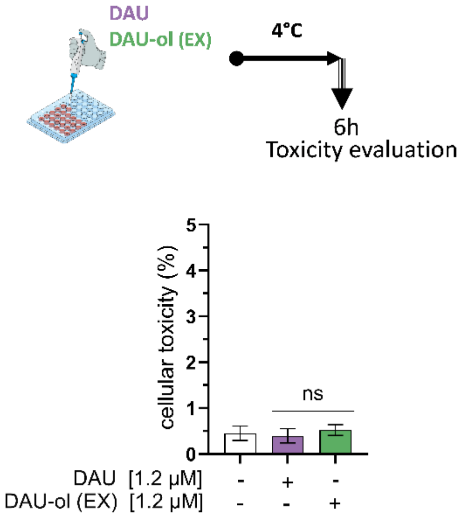

Figure S4. Toxicity of daunorubicin (DAU) and exogenously administered daunorubicinol (DAU-ol (EX)) after 6 hour incubation of rat neonatal ventricular cardiomyocytes (NVCM) at 4 °C. LDH method was used to determine the toxicity. Data are presented as means ± SD ($n \geq 4$); one-way ANOVA, $p \leq 0.05$ Dunnett test.

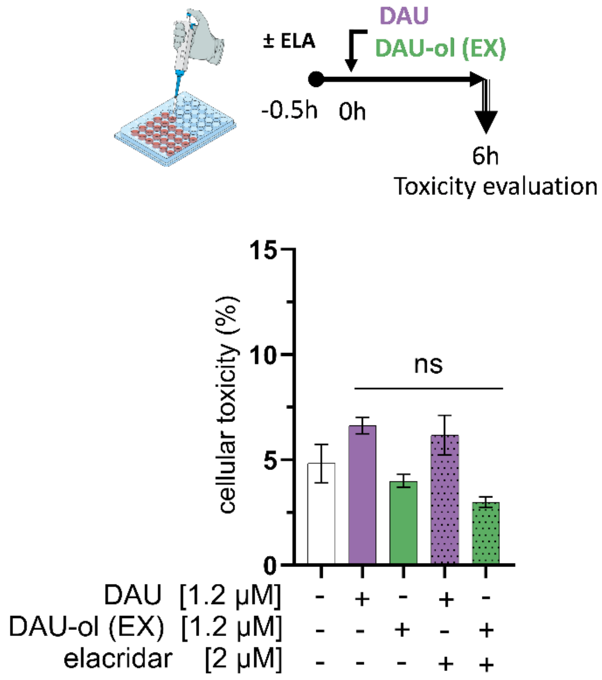

Figure S5. Toxicity of daunorubicin (DAU) and exogenously administrated daunorubicinol (DAU-ol (EX)) after 30 minutes of preincubation with P-gp inhibitor elacridar (ELA) followed by 6 hours incubation of rat neonatal ventricular cardiomyocytes (NVCM) with DAU or DAU-ol (EX) at 37°C. LDH method was used to determine the toxicity. Data are presented as means ± SD ($n \geq 4$); one-way ANOVA, $p \leq 0.05$ Dunnett test.

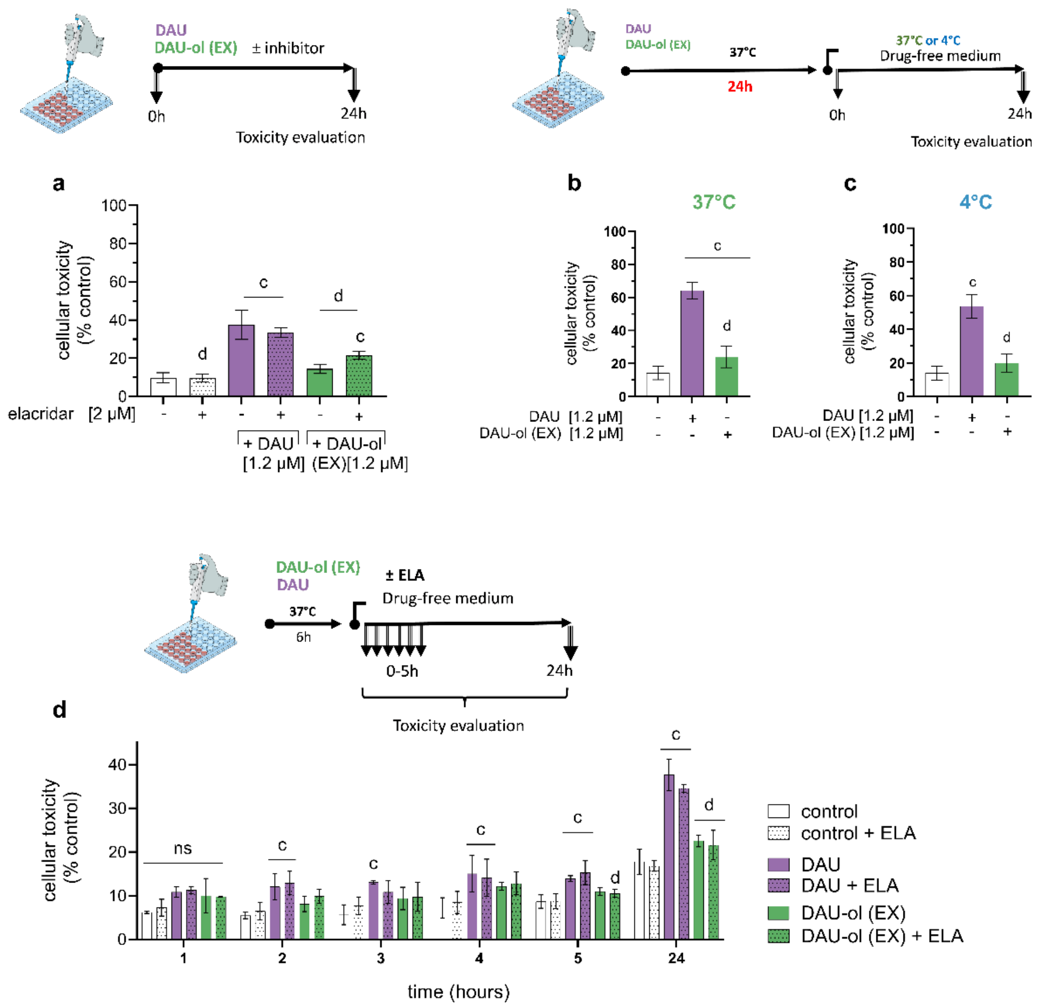

Figure S6. Toxicity of daunorubicin (DAU), exogenously administrated daunorubicinol (DAU-ol (EX)) and P-gp inhibitor elacridar (ELA) on isolated rat neonatal ventricular cardiomyocytes (NVCM) as a complementary assessments to efflux experiments. The cardiomyocytes were incubated for 24 hours with DAU, DAU-ol (EX) and/or ELA (2 μM) for determinations of their cardiotoxicity (**a**). Toxicity evaluation after 24 hours of incubation with DAU or DAU-ol (EX), which was followed by a 24-hour drug-free period at 37 °C (**b**) or 4 °C (**c**). Toxicity measurement after 6 hours of incubation with DAU or DAU-ol (EX) at 37 °C following a 1, 2, 3, 4, 5, or 24 hours drug-free period with or without ELA at 37 °C (**d**). LDH method was used to determine the toxicity. Data are presented as means ± SD ($n \geq 4$). Statistical significance (one-way ANOVA, $p \leq 0.05$; compared to: *c* – control, *d* – daunorubicin, *o* – exogenously administered daunorubicinol).

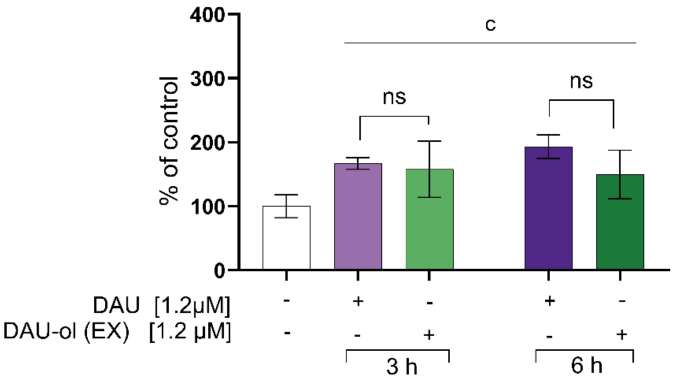

Figure S7. Comet assay for DNA damage detection. Rat neonatal ventricular cardiomyocytes (NVCM) were treated with 1.2 µM daunorubicin (DAU) or exogenously administrated daunorubicinol (DAU-ol (EX)) for 3 or 6 hours. Then, the single-cell gel electrophoresis was performed according to the previously published protocol (Olive and Banath 2006). Briefly, NVCM were plated in a 24-well plate (0.4 × 10^6 cells/well). After the drug treatment, NVCM were detached by accutase (150 µL, 15 min, RT, gentle agitation) and mixed with 250 µL ice-cold PBS. The suspension (20 µL) was mixed with 60 µL of 1 % low melting point agarose, mounted onto the microscope slide, and lysed overnight (2.5 M NaCl, 0.1 M EDTA, 100 mM Tris, 1 % Triton, pH 10). The next day, after alkaline unwinding (40 min on ice, 0.3 M NaOH), electrophoresis (300 mA, 14 V) ran for 30 min on ice, after neutralisation (0.4 M Tris, pH 7.5, 3 × 5 min). DNA in the mini gels were stained with ethidium bromide (0.1 µg/mL), documented (Nikon Eclipse Ti-E, Andor Zyla 5.5) and analysed with TriTek CometScore Freeware v1.5 for Windows. Data are expressed as percentage of the control and presented as means ± SD ($n \geq 4$). Statistical significance: one-way ANOVA, $p \leq 0.05$ Dunnett test (compared to *c* – control) and paired parametric t-test.

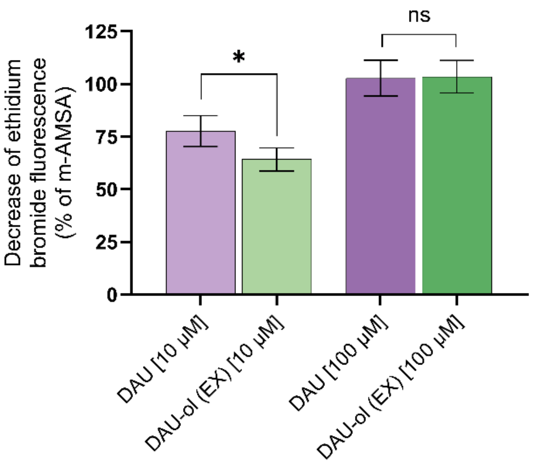

Figure S8. DNA intercalation assessed as ethidium bromide displacement. Daunorubicin (DAU) and exogenously administrated daunorubicinol (DAU-ol (EX)) were examined using an ethidium bromide displacement fluorescence assay (Fortune and Osheroff 1998; Matias-Barrios et al. 2021). Salmon sperm DNA (100 µg/mL) and ethidium bromide (2 µg/mL) were incubated (10 min, RT) either alone (negative control), with m-amsacrine (m-AMSA; positive control), or DAU or DAU-ol (EX), and the fluorescence emission of ethidium bromide in complex with DNA was measured (λ_{max} = 595 nm, excitation wavelength 546 nm). Data are expressed as relative to m-AMSA (mean ± SD; $n \geq 3$). Statistical significance: parametric t-test ($p \leq 0.05$).

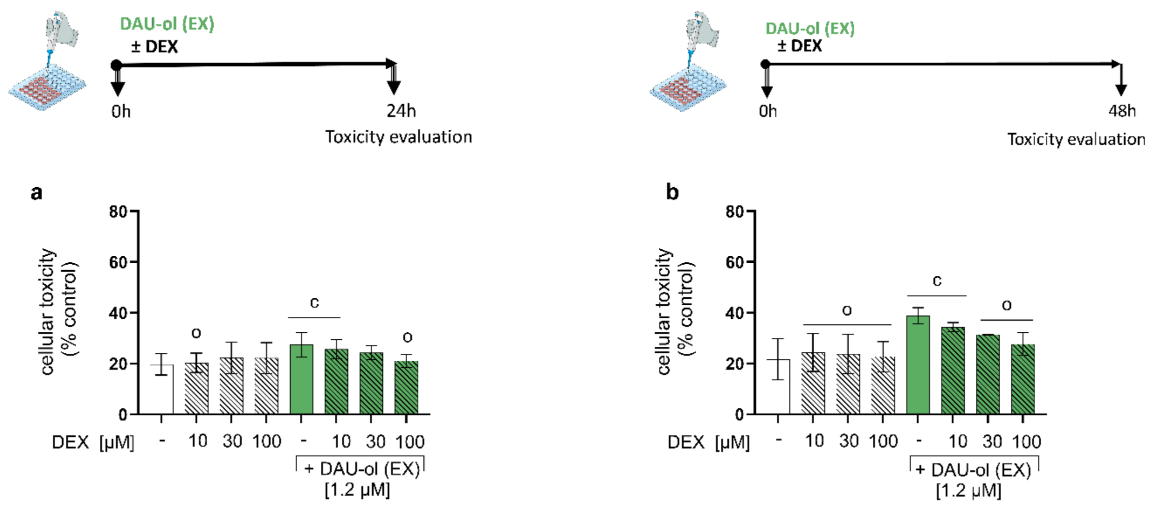

Figure S9. Effects of dexrazoxane (DEX) on cardiotoxicity induced by exogenously administrated daunorubicinol (DAU-ol (EX)) in rat neonatal ventricular cardiomyocytes (NVCM). Toxicity was evaluated by nucleic acid stain assay as an increased fluorescence signal. NVCM were incubated with DEX alone or in combination with DAU-ol (EX) for 24 hours (**a**) or 48 hours (**b**). Data are presented as means ± SD ($n \geq 4$). Statistical significance: one-way ANOVA, $p \leq 0.05$ Dunnett test (compared to *c* – control, *o* – exogenous daunorubicinol).

Supplementary References

Fortune JM, Osheroff N (1998) Merbarone inhibits the catalytic activity of human topoisomerase IIalpha by blocking DNA cleavage. J Biol Chem 273(28):17643-50 doi:10.1074/jbc.273.28.17643

Legrand C, Bour JM, Jacob C, et al. (1992) Lactate dehydrogenase (LDH) activity of the cultured eukaryotic cells as marker of the number of dead cells in the medium [corrected]. J Biotechnol 25(3):231-43 doi:10.1016/0168-1656(92)90158-6

Matias-Barrios VM, Radaeva M, Ho CH, et al. (2021) Optimization of New Catalytic Topoisomerase II Inhibitors as an Anti-Cancer Therapy. Cancers (Basel) 13(15) doi:10.3390/cancers13153675

Olive PL, Banath JP (2006) The comet assay: a method to measure DNA damage in individual cells. Nat Protoc 1(1):23-9 doi:10.1038/nprot.2006.5
